# Current clinical applications and future developments of platinum-based anticancer drugs

**DOI:** 10.1007/s00775-026-02172-x

**Published:** 2026-09-10

**Authors:** Maria S. Kamusheva, Hristo P. Varbanov

**Affiliations:** 1 Faculty of Pharmacy, Medical University of Sofia, Sofia, Bulgaria; 2 Institute of Pharmacy – Pharmaceutical Chemistry, University of Innsbruck, Innsbruck, Austria; 3 Medical College, Trakia University – Stara Zagora, Stara Zagora, Bulgaria

**Keywords:** Pt-based drugs, Oncology, Drug utilization, Chemotherapy, Clinical trials

## Abstract

The study evaluated the current patterns of guideline-recommended clinical use, real-world utilization trends, and translational development of platinum-based (Pt-based) drugs through a comparative analysis of national and international pharmacotherapeutic guidelines, national drug utilization data, clinical trials, and emerging research directions. Cisplatin, carboplatin and oxaliplatin continue to play central roles across multiple malignancies and remain widely incorporated into recommended treatment protocols. Across all five guideline sources evaluated, Pt-based therapies were recommended in more than half of the 18 ICD-O topographic categories, with guideline coverage ranging from 55.6% to 83.3%. Real-world utilization data, using Bulgaria as a case study, indicated sustained and increasing use of Pt-based therapies between 2021 and 2024, with oxaliplatin showing the greatest increase. Current clinical trials focus primarily on optimizing established Pt-based regimens through combinations with immunotherapeutics and emerging drug candidates within increasingly personalized multimodal treatment strategies. In parallel, research efforts are directed toward the development of new Pt complexes, exploring next-generation approaches such as multifunctional Pt(IV) prodrugs and Pt–N–heterocyclic carbene compounds, as well as advanced drug-delivery systems, reflecting continued innovation in the field. Overall, Pt-based drugs remain essential in cancer therapy and continue to represent a dynamic area of research in biological inorganic chemistry and translational oncology.

**Graphical Abstract:**

**Figure:**
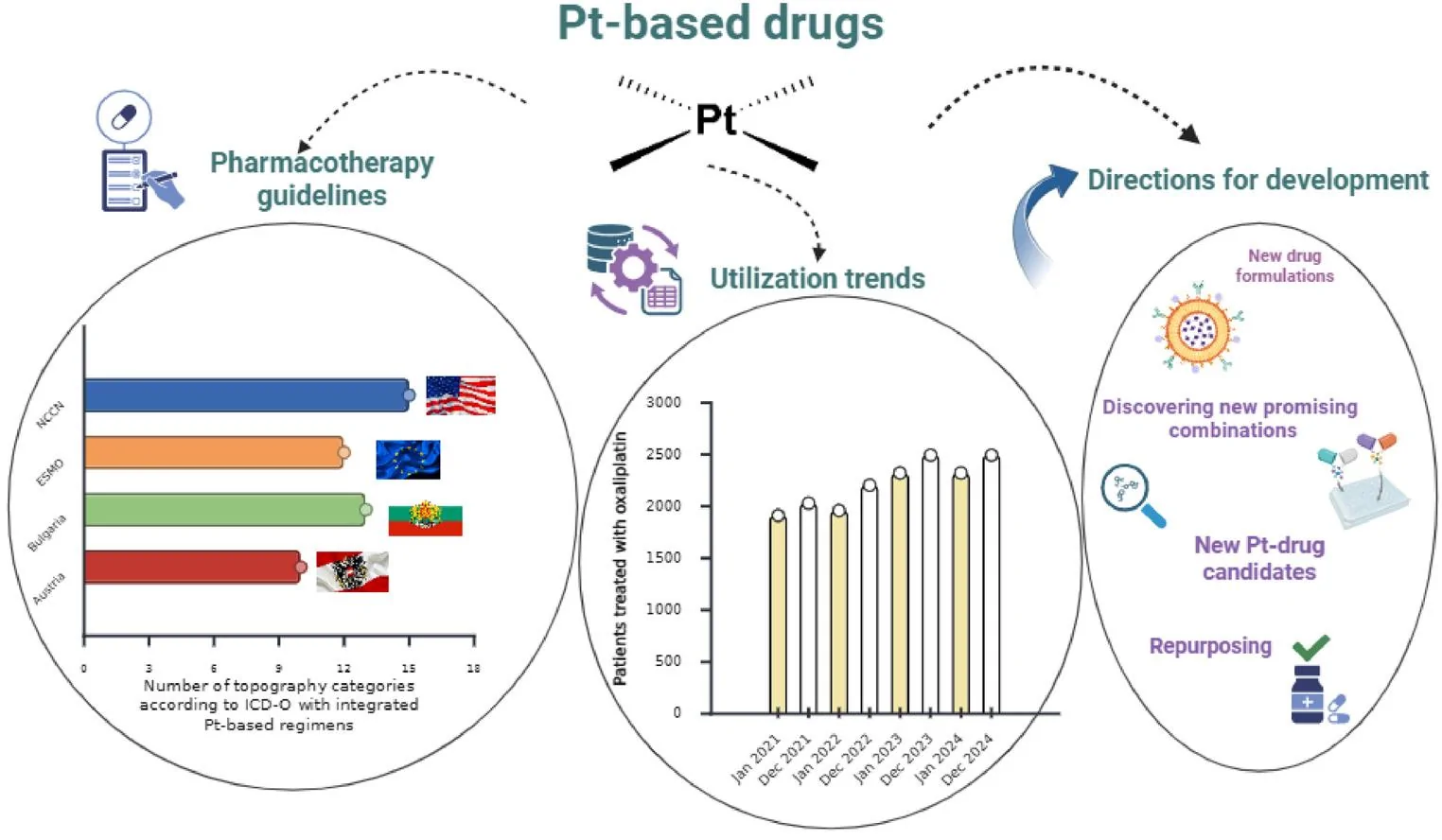

**Supplementary Information:**

The online version contains supplementary material available at https://doi.org/10.1007/s00775-026-02172-x.

## Introduction

Cancer comprises a diverse group of diseases fundamentally driven by genetic alterations, including mutations affecting three main classes of genes involved in tumorogenesis [1–3]. Hanahan et al. differentiated several hallmarks of human tumors such as reprogramming of energy metabolism and evading immune destruction used as a basis for further development of innovative anticancer agents [2]. According to data published by the World Health Organization (WHO) in 2022, 19,976,499 new cancer cases and 9.7 million cancer-related deaths were reported worldwide, with lung (12.4%), breast (11.5%) and colorectal (9.6%) cancers representing the most common malignancies [4]. According to a recent population-based study including 36 cancer types from 185 countries, the number of new cases is expected to exceed 35 million in 2050, with the most significant increase of the incidence expected in countries with low and medium human development index (HDI) [5].

Despite the availability of complex treatment modalities (surgery, radio-, chemo-, hormone-, immunotherapy) and continuous efforts in drug development and clinical research, the global burden of cancer remains substantial [4]. In the search for new therapies breakthroughs in chemistry and pharmaceutical sciences often emerge from coincidental discoveries [6]. Various methods for cancer treatment have been tested starting with low doses of radiation, immunotherapy, and synthetic hormone therapy. This progression followed the discovery of the first chemotherapeutic agent, mustine, and continued with the development of drugs such as anthracyclines, mercaptopurine, fluorouracil, cyclophosphamide, tamoxifen, vinca alkaloids, and others [7–9].

**Fig. 1:**
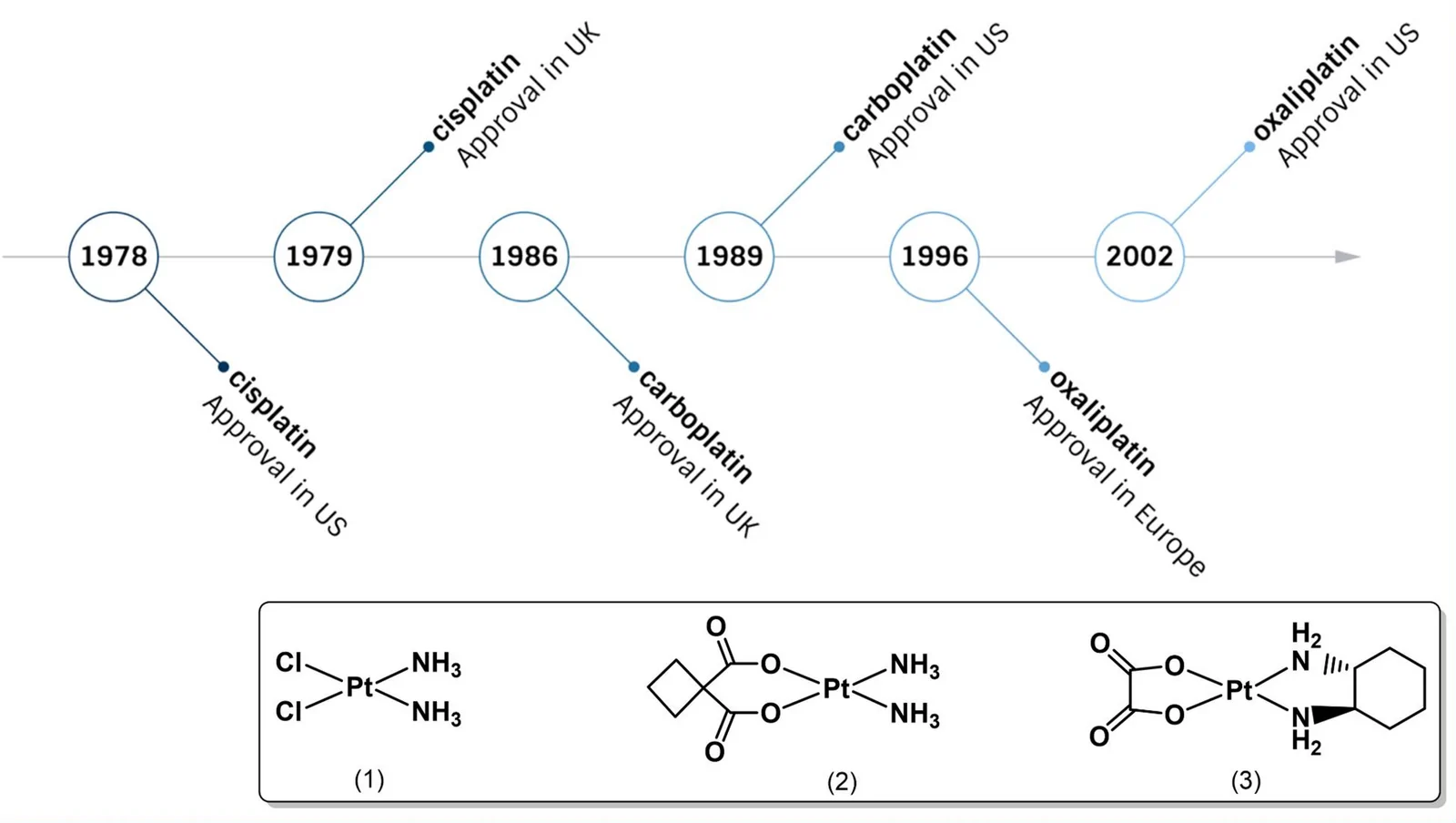
Platinum-based anticancer drugs approved for clinical use worldwide: cisplatin (1), carboplatin (2), and oxaliplatin (3), together with their years of regulatory approval

Platinum-based (Pt-based) drugs including cisplatin, carboplatin, and oxaliplatin (Fig. 1), have played a pivotal role in cancer therapy as they have high activity against several tumors [10, 11]. Their primary mechanism of action involves the formation of DNA crosslinks that disrupt DNA replication and transcription, leading to cancer-cell apoptosis [10, 12]. Pt-based drugs have a long history back in the years till now, beginning with Barnett Rosenberg’s discovery in 1965 of cisplatin’s effects on the morphology and growth of *Escherichia coli* [13].

Following its approval in the late 1970s, cisplatin (*cis-diamminedichloridoplatinum(II); cis*-[Pt(NH_3_)_2_Cl_2_]) became the first widely used metal-based anticancer drug and an essential component of treatment regimens for numerous malignancies such as lymphoma, sarcomas, mesotheliomas, neuroblastomas, ovarian, testicular, lung, head and neck cancers, etc. [14–16]. Considering its severe side effects (for example nephro-, oto-, hepatotoxicity) and resistance in certain cancer cell types, the need for new analogues with better toxicological and pharmacological profile had been raised [17–21].

One such analogue is carboplatin (*diammine(1*,*1- cyclobutanedicarboxylato)platinum(II*)), which development was led by professor Kenneth R. Harrap and his team in the late 1970s and introduced into clinical practice in the 1980s. Compared with cisplatin, carboplatin demonstrated similar therapeutic outcomes and less side effects in certain cancer types (mainly in ovarian and small cell lung cancer) [19, 22]. The widespread clinical implementation of carboplatin was further facilitated by the introduction of the Calvert formula, which enabled individualized dosing based on patients’ glomerular filtration rate (GFR) and reduced the risk of severe thrombocytopenia [23].

Subsequently, oxaliplatin ((*1R*,*2R)-1*,*2-diaminocyclohexaneoxalatoplatinum(II)*) entered the market in Europe and the United States (US) during the late 1990s and early 2000s. Compared with cisplatin, it demonstrated reduced nephrotoxicity and activity against cisplatin-resistant tumors, and became the first Pt-drug indicated for the treatment of colorectal cancer [15, 24].

In addition to these globally established platinum compounds, several other Pt-based anticancer agents have received regional approval, including nedaplatin (Japan, 1995), heptaplatin (South Korea, 1999), lobaplatin (China, 2003), miriplatin (Japan, 2009), and dicycloplatin (China, 2012) [15, 25–27]. Clinically, nedaplatin has been used in Japan for head and neck cancer, small cell lung cancer, non-small cell lung cancer, esophageal cancer, bladder cancer, testicular tumor, ovarian cancer and uterine cervix cancer [28, 29]; heptaplatin has been primarily indicated for advanced gastric cancer in South Korea [30]; and lobaplatin has been approved in China for small-cell lung cancer, metastatic breast cancer, and chronic myeloid leukemia [31]. Miriplatin represents a more specialized formulation indicated for hepatocellular carcinoma [32–34], whereas dicycloplatin has been clinically investigated in China, primarily in patients with lung cancer [35, 36].

Despite their considerable clinical effectiveness, Pt-based drugs are associated with a number of challenges, such as toxicity, drug resistance, and the high costs and inconvenience related to their administration. These costs are mainly associated with the need for inpatient care, trained nursing staff, specialized medical devices, and equipment [37–39]. In order to cope with these issues, many efforts are directed to synthesize new platinum complexes with improved physicochemical properties or to develop better formulations [11].

Although newer cancer therapies are increasingly targeted and potentially less toxic, Pt-based agents remain a major component of anticancer therapy despite their relatively simple chemical structures and limitations related to toxicity and resistance. It is frequently stated in the literature, usually based on older review articles or drug reference books such as *Martindale*, that more than 50% of cancer patients receive a platinum compound as part of their treatment. To investigate actual patterns of platinum drugs usage across different cancer types in a clinical setting, Armstrong‑Gordon et al. examined chemotherapy regimens in a single Australian hospital in 2014–2015 and found that 46% of 156 cancer patients received a platinum drug [40]. Similarly, Um et al. reported that platinum agents were recommended in 25% of chemotherapy protocols according to Australian eviQ guidelines [41].

Although Pt-based drugs remain central to modern oncology, comprehensive evaluations of their current utilization patterns across malignancies and their alignment with international pharmacotherapeutic guidelines remain limited.

Accordingly, this study aims to address the following primary research question: “What are the current patterns of guideline-recommended use of globally approved Pt-based drugs (cisplatin, carboplatin, and oxaliplatin) across national and international guidelines, and what trends in their clinical utilization are observed at the national level?”. To complement this analysis, the study also evaluates current clinical trial activity involving both globally and regionally approved Pt agents, as well as newer investigational Pt modalities, providing additional insight into their contemporary clinical positioning. Finally, the article explores scientific developments that may expand the role of Pt-based therapeutics in oncology.

## Methods and materials

### Regulatory status of Pt-based drugs

Regulatory information was extracted from publicly available regulatory databases to identify Pt-based anticancer drugs authorized for marketing and available in the USA, the European Union (EU), Austria, and Bulgaria. A list of clinically relevant Pt-based agents was compiled through a literature review and pharmacological classification of antineoplastic agents.

For the USA, marketing authorization data and the number of approved medicinal products were retrieved from the US Food and Drug Administration (FDA) National Drug Code (NDC) Directory [42]. For the EU, regulatory status and orphan drug designation information were identified using the European Medicines Agency (EMA) databases, including the Community Register of Medicinal Products and the Orphanet/EMA orphan designation register [43]. National regulatory data for Austria were obtained from the Austrian Medicines and Medical Devices Agency (BASG) medicinal product register, while data for Bulgaria were retrieved from the Bulgarian Drug Agency (BDA) medicinal product register [44, 45].

The database searches were conducted in August 2025. For each Pt-based drug, the following information was extracted: international non-proprietary name (INN), number of authorized medicinal products, country of the marketing authorization holder, and year of marketing authorization. Generic products were defined as medicinal products containing the same active substance, strength, pharmaceutical form, and route of administration as the reference medicinal product after patent expiration. The collected data were compiled and compared across the analyzed jurisdictions to assess differences in regulatory availability and market presence of Pt-based medicinal products. In addition, orphan drug designations related to platinum-containing therapies were identified and summarized based on EMA records.

### Clinical guidelines analysis

Current clinical guidelines and oncology treatment protocols were reviewed to assess guideline-recommended clinical use, defined as the formal positioning of Pt-based medicines within recommended treatment pathways. Because clinical guidelines inform prescribing decisions and, in some healthcare systems, are linked to public reimbursement requirements, the extent of guideline coverage was considered an indicator of the recognized clinical role of Pt-based therapy. It was not interpreted as a direct measure of patient-level exposure or actual prescribing frequency.

The analysis focused on the globally approved Pt-drugs, cisplatin, carboplatin, and oxaliplatin (Fig. 1), and assessed their recommended use by cancer type, line of therapy (first or second line), and treatment modality (monotherapy or combination therapy). These agents were selected because of their broad multinational approval and consistent representation across the guidelines, jurisdictions, and utilization datasets examined.

The evaluation was conducted according to established clinical standards defined by the European Society for Medical Oncology (ESMO), the U.S. National Comprehensive Cancer Network (NCCN), the Bulgarian pharmacotherapeutic guidelines, and the legally approved Austrian oncology chemotherapy protocols [46–49]. Previously published data from the Australian eviQ protocols [41] were additionally incorporated into the analysis to allow comparison with the four directly reviewed guideline systems.

All guidelines were systematically analyzed to determine the indications for Pt-based agents with respect to the categories defined above, and relevant data was extracted into tables. To facilitate comparison across guidelines, cancer types were harmonized using the WHO Family of International Classifications (WHO-FIC) Maintenance Platform, specifically the International Classification of Diseases for Oncology (ICD-O) linearization. The ICD-O system codes neoplasms by topography (C00–C80), aligned with ICD-10, and by morphology, reflecting histologic type and biological behavior [50]. Mapping to ICD-O topography codes enabled standardized comparison across guidelines, following the anatomical groupings: C00–C14 (lip, oral cavity, and pharynx); C15–C26 (digestive organs); C30–C39 (respiratory system and intrathoracic organs); C40–C41 (bones, joints, and articular cartilage); C42 (hematopoietic and reticuloendothelial systems); C44 (skin); C47 (peripheral nerves and autonomic nervous system); C48 (retroperitoneum and peritoneum); C49 (connective, subcutaneous, and other soft tissues); C50 (breast); C51–C58 (female genital organs); C60–C63 (male genital organs); C64–C68 (urinary tract); C69–C72 (eye, brain, and other parts of the central nervous system); C73–C75 (thyroid and other endocrine glands); C76 (other and ill-defined sites); C77 (lymph nodes); and C80 (unknown primary site).

In addition, the proportion of cancer types for which Pt-based regimens were recommended was calculated as a percentage of the 18 ICD-O anatomical categories considered. Cancer types were mapped to the ICD-O classification to assess the distribution of Pt-based chemotherapy across tumor types and anatomical localizations in different geographic regions

$$\:Index\:per\:guideline\:\left(\%\right)=\frac{n\:}{18}\times\:100.$$

where *n* represents the number of anatomical categories, according to the ICD-O classification, for which Pt-based regimens are recommended within a given guideline; 18 represents the total number of anatomical categories included in the analysis. The Index ranges from 0% to 100%.

### Analysis of drug utilization

Retrospective qualitative and quantitative analyses of Pt-based drugs utilization were performed using Bulgaria as a case study, encompassing both national- and hospital-level evaluations. Bulgaria was selected considering several factors: it reports high rates of incidence and preventable mortality of cancer and other chronic diseases (more than two times higher than the average value for the EU) [51]; the low levels of cancer screening and preventive physicians’ visits in the country, and the availability of publicly accessible pharmaceutical and reimbursement data sources suitable for drug utilization analysis.

The first objective was to examine the patterns of Pt-based agents’ usage through a retrospective study based on evaluation of national drug utilization data. This analysis was conducted at the national level using data from the Bulgarian National Health Insurance Fund (NHIF). Key metrics for this evaluation included the number of patients treated, the number of drug packages dispensed and the most common neoplasms for which Pt-drugs were used for a 4-year period of time (2021–2024). The data in the NHIF database regarding diseases are presented according to the International Classification of Diseases, 10th revision (ICD-10) [52].

The other objective was to assess the prescribing patterns and utilization of Pt-based agents in a hospital setting, where their use is limited, as the malignancies treated there are not typically managed with Pt-based therapies as first line treatment. To address this objective, a retrospective analysis on the utilization of Pt-based drugs was conducted at the Specialized Hospital for Active Treatment of Hematological Diseases (SHATHD), Sofia, Bulgaria. This hospital functions as the national reference center for the diagnosis, treatment, and follow-up of hematological malignancies, receiving referrals from healthcare facilities across the country. The hospital’s pharmacy department maintains a systematically organized, comprehensive database documenting all dispensed drug units, linked to patient treatment records. This ensured high accuracy, completeness, and traceability of the data, allowing for robust quantitative analysis and integration into the national-level assessment of Pt-based agent utilization. Patient medical records for a one-year period (2024) were reviewed to extract demographic information, year of diagnosis, data on prescribed agents, and change in therapeutic regimens. Patient data were processed and systematized using MedCalc^®^ statistical software, as descriptive statistics were applied to summarize the variables. Moreover, data on the total number of treated oncohematology patients and their corresponding diagnoses, as reported by the same hospital per month, were extracted from the NHIF website. The proportions of oncohematology patients receiving Pt-based therapy (*P*_*p*_) and the respective diagnoses (*P*_*d*_) relative to all oncohematology patients and diagnoses treated at the hospital were subsequently calculated per month.

$$P_p({ \% })={\frac{{n}_{p}}{{N}_{p}}}\times100.$$

where *n*_*p*_ is the number of oncohematology patients treated with Pt-based drugs at the hospital of interest, and *N*_*p*_ is the total number of oncohematology patients treated at the hospital of interest

$$P_d({ \% })={\frac{{n}_{d}}{{N}_{d}}}\times100.$$

where *n*_*d*_ is the number of oncohematology diagnoses for which Pt-based drugs are prescribed at the hospital of interest, and *N*_*d*_ is the total number of oncohematology diagnoses treated at the same hospital.

### Clinical trials analysis

The analysis focused on identifying relevant clinical trials (CTs) that provide insight into current trends in the clinical exploration of Pt-based drugs. It was limited to studies registered in the *ClinicalTrials.gov* database with an *“Active*,* not recruiting”* recruitment status and a study start date between 01/01/2022 and 31/12/2025. Although this registry includes studies conducted in numerous countries worldwide, it does not provide comprehensive coverage of all trials globally, as some studies may be registered exclusively in other national or regional registries. Consequently, the geographical distribution of the retrieved records may be affected by differences in registry coverage and registration practices.

The Pt-based drugs considered comprised three groups: (1) globally approved agents, including cisplatin, carboplatin, and oxaliplatin; (2) regionally approved agents, including dicycloplatin, heptaplatin, lobaplatin, miriplatin, and nedaplatin; and (3) clinical candidates without regulatory approval that have previously entered clinical development, including picoplatin, iproplatin, satraplatin, phosplatin (PT-112), BTP-114, and lipoplatin (nanoplatin). This group included both older clinical candidates and more recent Pt-based modalities, as some compounds that had previously entered clinical trials may have subsequently been investigated in newer trials meeting the predefined eligibility criteria.

A search was conducted to identify relevant CTs, with Boolean operators (AND, OR) applied to refine the search. All applicable records were exported to Mendeley Reference Management Software for organization and screening. Records were assessed for eligibility based on predefined inclusion and exclusion criteria. Eligibility criteria included: (1) interventional study design; (2) inclusion of Pt-based drugs among the study interventions; (3) focus on the safety and efficacy of Pt-based treatment regimens, including the addition of new drug candidates, or on improving their therapeutic profile for cancer indications; and (4) studies conducted in any clinical-trial phase (1–4). Studies were excluded if they (1) did not involve Pt-based drugs; (2) lacked sufficient relevant information for the purposes of the present analysis; (3) did not address the relevant research questions; or (4) contained insufficient information for evaluation.

### Literature search for trend analysis of Pt-based drugs research

A literature search was conducted in PubMed to identify all published studies on: (1) Pt-based anticancer compounds; (2) Pt–NHC compounds; (3) multifunctional platinum(IV) complexes; (4) repurposing strategies involving Pt-drugs, and (5) new drug-delivery systems for Pt-based drugs. Searches covered the entire period available in the database, combining controlled vocabulary (MeSH terms) and free-text keywords searched in the Title/Abstract fields, according to the formulated objectives. Searches related to repurposing and drug-delivery dosage forms were limited to the period 2022–2025 to focus on the latest evidence, reflecting advances and innovations in these fields. The complete search strategies are provided in the Supplementary information (Table S1). For searches 2–5, retrieved records were manually screened to exclude non-relevant articles and retain only studies that met the predefined relevance criteria. Compound annual growth rates (CAGR) were calculated for searches 1–3 to assess the rate of increase in the number of published studies over the defined period.

$$\:\mathrm{C}\mathrm{A}\mathrm{G}\mathrm{R}\:\left(\mathrm{\%}\right)={[\:\left(\frac{\mathrm{N}\mathrm{f}}{\mathrm{N}\mathrm{i}}\right)}^{1/\mathrm{t}}-1]\times100$$

where *N*_*f*_ *–* final year in the observed period, *N*_*i*_ *–* initial year in the observed period, *t –* number of years in the observed period.

It should be noted that these searches do not intend to provide a comprehensive literature review, but rather to draw the general trend and major research directions in the field of Pt-based drugs research.

## Results and discussion

### Regulatory status and market availability of Pt-based drugs

Analyzing the American and selected European markets, and focusing on the globally authorized Pt-drugs (cisplatin, carboplatin, and oxaliplatin), it is evident that numerous generics entered the market following patent expirations. Reflecting the size of the USA market, there are currently 25, 46 and 57 generics available for cisplatin, carboplatin, and oxaliplatin, respectively (Table 1). In contrast, European countries like Austria and Bulgaria have far fewer generic alternatives, with oxaliplatin having the highest number among the three drugs. Despite ongoing efforts to develop new platinum compounds, cisplatin, carboplatin, and oxaliplatin remain the only Pt-based drugs widely authorized across major global regulatory markets. Several other Pt-drugs have received regional approval in Asian countries, but lack broad global authorization.

In the EU, in addition to their established indications, several therapies, including Pt complexes have been authorized and received orphan designation status. These include carboplatin/sodium valproate for glioma (in 2018), carboplatin for glioma (in 2024), liposomal cisplatin for pancreatic cancer (in 2007), lipid-complexed cisplatin for osteosarcoma (in 2013), cisplatin for malignant mesothelioma (in 2016), and cisplatin for glioma (in 2024). In 2007 an orphan designation status was given also to picoplatin for small cell lung cancer, but it was withdrawn on request of the sponsor [53]. These orphan designations indicate the scientific efforts to expand the therapeutic use of Pt-based drugs also for rare cancers and treatment-resistant malignancies, highlighting their repurposing potential.

**Table 1:** Pt-based medicinal products currently authorized for sale in the USA, Austria and Bulgaria

INN	USA			Bulgaria			Austria		
	Number of MA products	MA holder-country	MA date	Number of MA products	MA holder-country	MA date	Number of MA products	MA holder-country	MA date
Carboplatin	46	USA, Canada, India, Israel	2004, 2006, 2007, 2009, 2014, 2015, 2016, 2017, 2020, 2022, 2023, 2024	3	Austria	1997, 1999, 2010	3	Netherlands, Austria	1995, 1998, 2010
Cisplatin	25	USA, Austria, Canada, India, Israel	2000, 2012, 2015, 2016, 2017, 2019, 2023, 2024	6	Austria, Poland	2005, 2011	5	Austria; Netherlands; Portugal	1987, 1994, 2011, 2022, 2015
Oxaliplatin	57	USA, India, Israel, Switzerland, France, Canada, China	2009, 2010, 2014, 2015, 2016, 2017, 2018, 2019, 2020, 2024	15	Bulgaria, Austria, Poland, Germany, Iceland, Luxemburg, Spain, UK, France	2008, 2011, 2012, 2017	6	Netherlands; Austria; Portugal	2006, 2010, 2011, 2012, 2022

**Abbreviations**: MA – marketing authorization; INN – international non-proprietary name

Number of reimbursed medicinal products (MPs) in Bulgaria: cisplatin – 1; oxaliplatin – 5; carboplatin – 1. For USA and Austria – N/A

### Implementation of Pt-based drugs in current oncology therapeutic guidelines

**Fig. 2:**
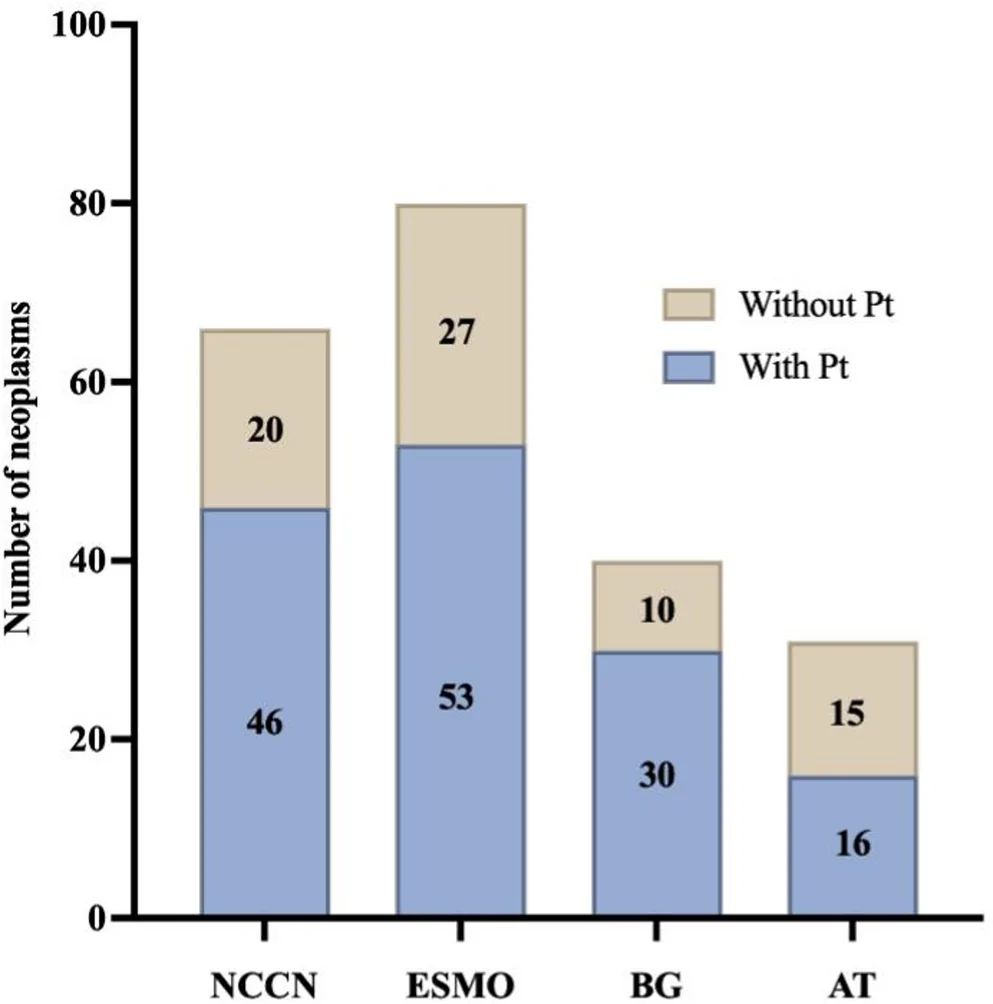
Number of neoplasms with Pt-based drug regimens (blue) in relation to the total number of neoplasms covered in the analyzed pharmacotherapeutic guidelines

**Fig. 3:**
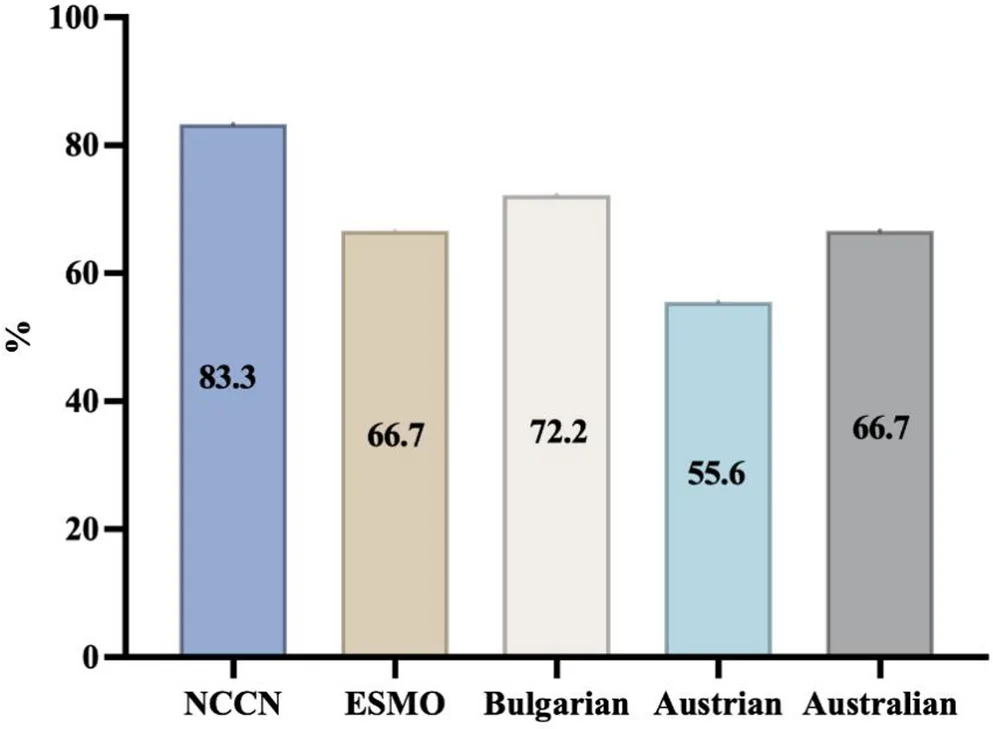
Percentage of cancer categories (harmonized using the ICD-O linearization classification) where Pt-based regimens are recommended in guidelines analyzed

Both the ESMO and the NCCN guidelines emphasize the strategic use of Pt-based drugs in various malignancies. For instance, the ESMO guidelines are organized into 13 main cancer categories: breast cancer; cancer of unknown primary; endocrine and neuroendocrine cancers; gastrointestinal; genitourinary; gynecological; hematological malignancies; head and neck cancer; hereditary syndromes; lung and chest tumors; melanoma and skin cancer; neuro-oncology; sarcoma and gastrointestinal stromal tumors [46, 47]. For each of these categories, multiple documents are available for specific subtypes, such as breast cancer in young women, early-stage breast cancer, and metastatic breast cancer (within the breast cancer category), various gastrointestinal cancers (anal, biliary, gastric, localized colon, metastatic colorectal, esophageal, pancreatic, and rectal cancers), etc. (Table S2). Among 80 documents across the different cancer types, Pt-based drugs are included in the therapeutic regimens in 53 (66.3%) of them, demonstrating their broad application (Fig. 2). Examples include adjuvant, neoadjuvant, and post-neoadjuvant systemic treatment in breast cancer (carboplatin plus taxanes); subsequent cytotoxic therapy for adrenocortical carcinoma amenable to complete remission (cisplatin plus etoposide); a second-line therapy for patients with advanced lung carcinoid refractory to or intolerant of other treatments; a standard of care in patients with advanced anal cancer cisplatin in combination with 5-FU/capecitabine, carboplatin or docetaxel-based combinations, etc. Mapped to ICD-O topography categories, Pt-based drugs are included in 12 of 18 categories (66.7%) in the ESMO guidelines. (Fig.3 and Table S6).

The NCCN guidelines cover a total of 66 different cancer types, of which 46 (69.7%) incorporate Pt-based therapy within treatment schemes for malignancies, such as anal carcinoma, B-cell lymphomas, bladder cancer, central nervous system (CNS), cervical, colon, gastric, head and neck, hepatocellular, kidney, non-small cell lung cancer, pancreatic adenocarcinoma and others (Fig. 2 and Table S3) [47]. In some cases, platinum doublets (e.g., cisplatin or carboplatin combined with paclitaxel or pemetrexed for non-small cell lung cancer) are recommended, often depending on the patient’s performance status and comorbidities. Mapped to ICD-O topography categories, Pt-based drugs are included in 15 out of 18 categories (83.3%), demonstrating their broad application across tumor types (Fig. 3 and Table S6).

The Bulgarian guidelines for oncological and hematological diseases were included in the analysis, given that clinical practices in Bulgaria are largely aligned with those outlined in major European and American guidelines (ESMO and NCCN) [48]. The guidelines cover 40 different cancer types, and Pt-based therapies are included in 30 of them (75.0%). Among these, cisplatin is the most frequently preferred agent, recommended in all 30 cancer types, followed by carboplatin (in 16 types) and oxaliplatin (in 8) (Table S4). Oxaliplatin is included in the protocols for gastric, pancreatic, hepatocellular, biliary and colorectal cancers, carcinoma of unknown primary, B-cell lymphoma and other lymphomas. Based on the ICD-O classification by topography categories, Pt-based drugs are integrated into the therapeutic approach in 13 out of 18 categories (72.2%)—a proportion similar to that observed in the ESMO guidelines (Fig. 3 and Table S6).

The Chemoprotocols.eu guidelines, which outline the most important and commonly used protocols for treating solid tumors in Austria, indicate that Pt-based drugs are included in 27% of chemotherapy protocols and 25% of antibody-based regimens, primarily as part of combination therapies (Table S5) [49]. The guideline includes 31 different cancer types as Pt-based therapy is incorporated in 16 out of them (51.6%) (Fig. 2). Out of 40 Pt-based regimens, cisplatin is present in 16 (mainly for cancers affecting digestive organs, male genital organs, connective, subcutaneous and other soft tissues), carboplatin in 11 (mainly for respiratory system, breast and female genital organs) and oxaliplatin in 11 (for digestive organs). Based on the ICD-O classification by topography categories, Pt-based drugs are integrated into the therapeutic approach in 10 out of 18 categories (55.6%) - a proportion that is lower than the other analyzed guidelines (Fig.3 and Table S6).

A 2019 study of the EviQ chemotherapy guidelines demonstrated that Pt-based drugs are also an integral part of treatment protocols in Australia, being included in 119 of 470 protocols for 24 specific cancer types [41]. We reclassified these cancers under ICD-O codes C00–14, C15–26, C30–39, C40–41, C42, C44, C47, C49, C50, C51–58, C60–63, and C64–68. Collectively, these categories account for 66.7% of all ICD-O cancer groups (12 of 18 categories) (Fig. 3).

Overall, treatment protocols containing Pt-based drugs are recommended in more than half of the 18 ICD-O cancer topography categories, across all reviewed guidelines, highlighting the continued central role of platinum agents in modern oncology. The NCCN guidelines show the widest coverage of Pt-based therapies among cancer categories (83.3%), followed by the Bulgarian (72.2%) and the ESMO guidelines (66.7%), while the Austrian Chemoprotocols show lower, but still substantial coverage (55.6%). All guidelines consistently recommend Pt-based therapies for major tumor groups such as head and neck, digestive, respiratory, breast, genital, urinary, and endocrine cancers. Variability appears primarily in less common sites like bone, skin, peripheral nerve, and CNS malignancies (Fig. 3). These percentages reflect the breadth of platinum use across cancer categories and should not be interpreted as the proportion of patients receiving Pt-based therapy.

The ICD-O mapping enabled a standardized comparison across guideline systems but does not fully capture the biological and clinical heterogeneity within individual cancer types. For instance, in breast cancer, the clinical role of Pt-based agents cannot be interpreted solely by anatomical site. Treatment selection is determined by estrogen-receptor (ER), progesterone-receptor (PR), and HER2 status. In hormone receptor-positive disease (ER- and/or PR-positive), endocrine therapy is a central component of treatment, frequently combined with other targeted agents. HER2-positive disease is primarily treated with HER2-directed therapies combined with chemotherapy, which may include carboplatin. Triple-negative breast cancer (TNBC) is treated mainly with chemotherapy and, in eligible patients, immunotherapy; carboplatin is incorporated into selected neoadjuvant regimens for early TNBC [54, 55]. Therefore, the ICD-O topography mapping used in the present analysis reflects anatomical guideline coverage but does not replace receptor- and biomarker-based clinical stratification.

Across all analyzed regions, guidelines recommend careful monitoring of renal function and neurotoxicity when using Pt-based agents, and substitution between cisplatin and carboplatin is commonly advised based on patient tolerance.

### To what extent are they currently in use – a case with Bulgaria?

#### National-based data

Cancer epidemiology data in Bulgaria provide important context for understanding the utilization of Pt-based anticancer therapies. In 2022 and 2023, cancers of the digestive system were the most prevalent with 5,833 and 5,664 cases, respectively, followed by melanoma and other malignant neoplasms of the skin (4,376 and 4,459 cases); breast cancer (3,139 and 3,139 cases); cancers of the male genital organs (2,531 and 2,843 cases); cancers of the female genital organs (2,460 and 2,526 cases); and cancers of the urinary system (2,119 and 2,139 cases) [56, 57]. Prostate cancer was the most commonly diagnosed malignancy among men in 2022, followed by colorectal and lung cancer, while breast cancer ranked first among women, with colorectal cancer second [51]. In terms of mortality, lung, colorectal, breast, pancreatic, prostate, and stomach cancers accounted for the largest share of cancer-related deaths in 2023 [51, 57]. These patterns highlight the predominance of cancers for which Pt-based therapies are commonly indicated.

**Fig. 4:**
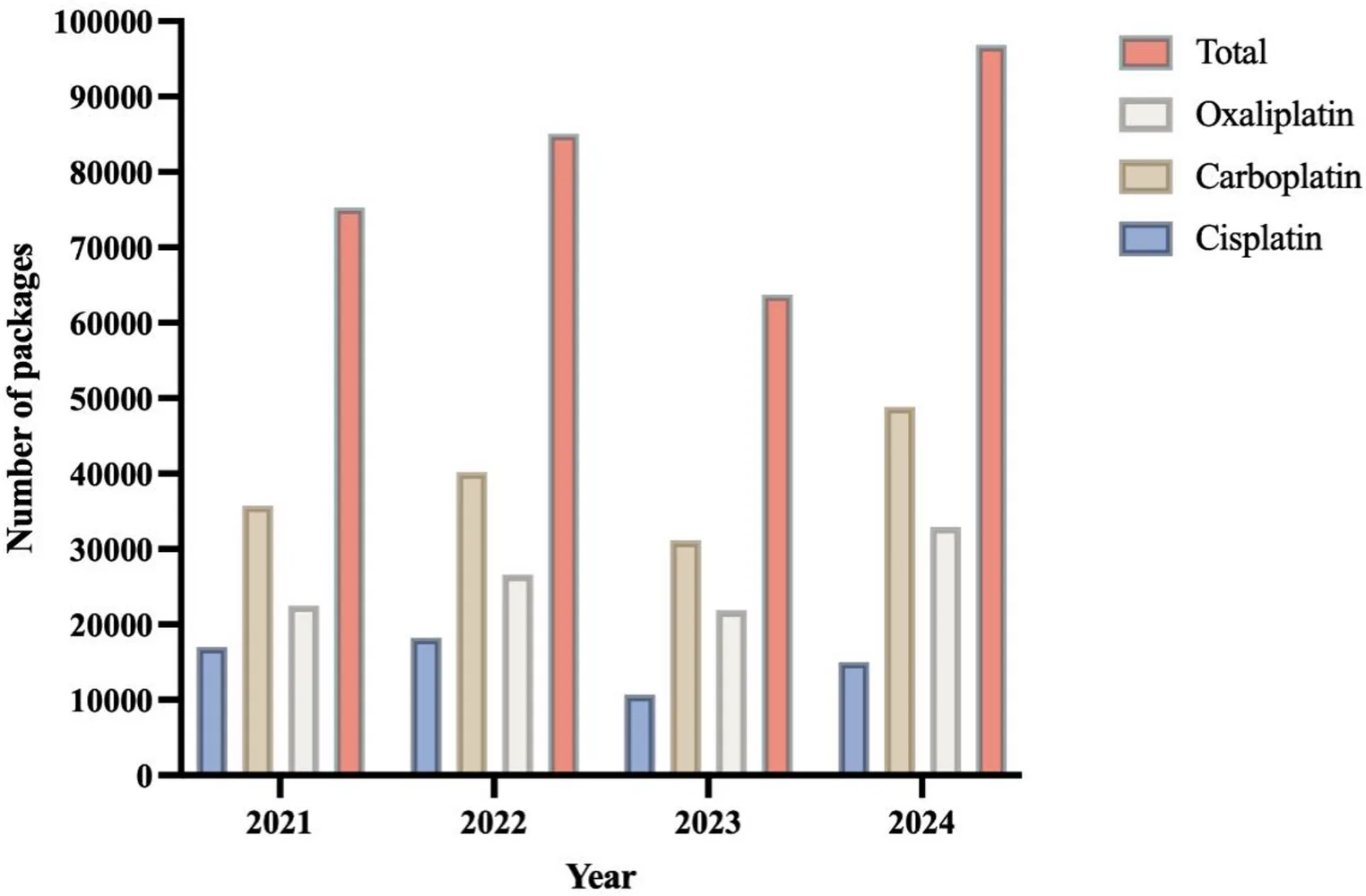
Annual number of reimbursed packages of Pt-drugs in Bulgaria for the period 2021–2024

**Fig. 5:**
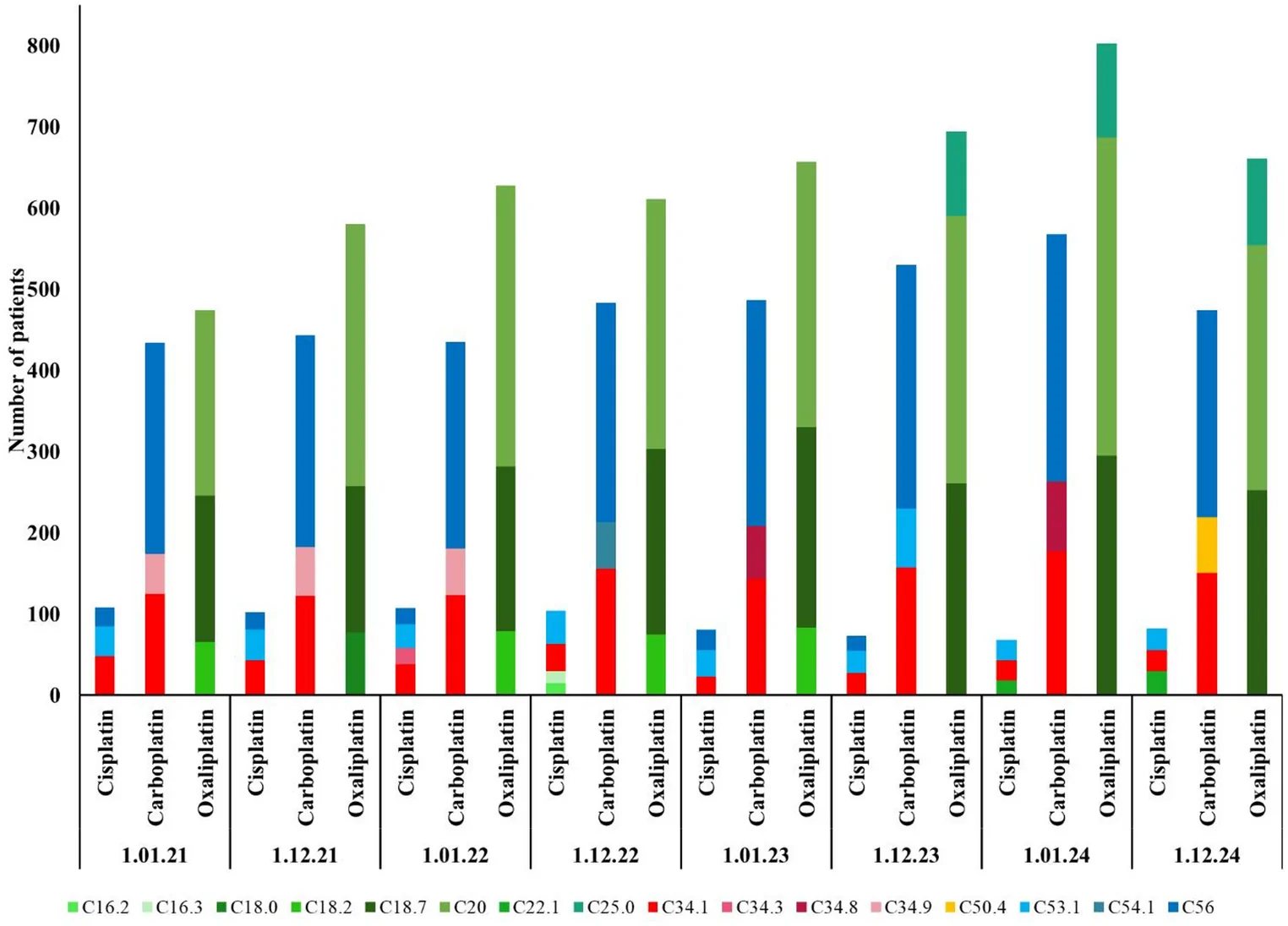
Top 3 cancers (classified according to ICD-O) treated with Pt-drugs in Bulgaria: January & December for the study period. *C15-C26 Digestive organs (green colors)*: C16.2 - Body of the stomach; C16.3 - Antrum of the pylorus; C18.0 - Cecum; C18.2 - Ascending colon; C18.7 - Sigmoid colon; C20 – Malignant neoplasm of the rectum; C22.1 - Carcinoma of the intrahepatic bile ducts; C25.0 - Head of the pancreas. *C30-C39 Respiratory system and intrathoracic organs (red colors)*: C34.1 - Upper lobe, bronchus or lung; C34.3 - Lower lobe, bronchus or lung; C34.8 - Lesion extending beyond the boundaries of one or more specified locations of the bronchus or lung; C34.9 - Bronchus or lung, unspecified location; *C50 Breast (yellow color)*: C50.4 - Malignant neoplasm (cancer) of the upper-outer quadrant of the breast; *C51-C58 Female genital organs (blue colors)*: C53.1 - Exocervix; C54.1 – Endometrium; C56 - Malignant neoplasm of the ovary. The codes are according to the International Classification of Diseases, Tenth Revision system

Consistent with the epidemiological profile, the utilization of Pt-based drugs in Bulgaria increased from 75,275 packages in 2021 to 96,840 in 2024 (Fig. 4 and Fig. S1). As shown in Fig. 5, Fig. S2 and Table S7, the main neoplasms for which Pt-drugs were reimbursed at the beginning and end of the observed period of time (01.2021–12.2024) include cancers of the digestive system (*rectum*,* sigmoid colon*,* ascending colon*,* cecum*,* pyloric antrum*,* head of the pancreas*,* carcinoma of the intrahepatic bile ducts)*, female genital organs *(ovary*,* exocervix*,* endometrium)*, respiratory system *(lower lobe*,* bronchus or lung*,* upper lobe*,* bronchus or lung*,* bronchus or lung*,* with unspecified localization)*, and breast. This aligns with the most common cancer localizations reported at the national level.

**Fig. 6:**
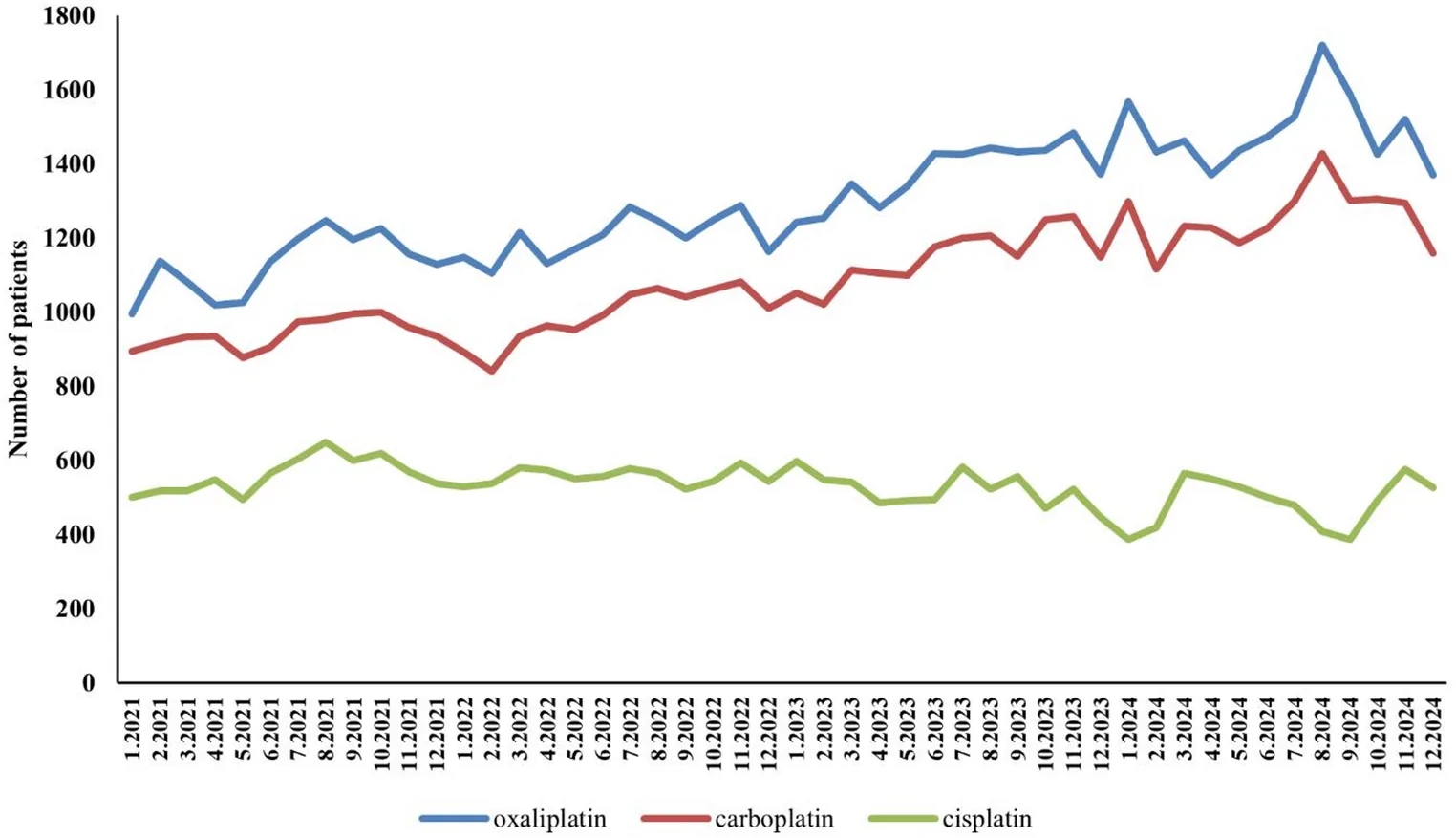
Number of health-insured patients per month (2021–2024) treated with Pt-based drugs in Bulgaria

As the number of people diagnosed with cancers affecting the digestive system, where oxaliplatin is a key component of standard treatment protocols, increases, the number of patients on this therapy and the consumed packages of oxaliplatin formulations also rise (Fig.6 and Fig. S1). Carboplatin consumption is also showing a positive trend, reflecting its main indications (for lung cancer and cancer of female genital organs), for which the total number of newly diagnosed patients during the observed period has also increased. Cisplatin and carboplatin are given for similar indications, while carboplatin is preferred by Bulgarian oncologists which is also visible from the graphs. This preference can be explained by its more favorable safety profile compared to cisplatin, particularly in higher-risk patient populations. Nevertheless, a more in-depth analysis based on real-world patient data would be beneficial to explain the trends.

#### Oncohematology hospital-based data

Although Pt-based chemotherapeutic agents are not considered first-line therapy for the majority of oncohematological diseases, they are administered in specific clinical scenarios, including refractory or relapsed disease, complex multimodal therapeutic regimens, and in patients with concomitant solid tumors. Inclusion of SHATHD in the present analysis provided a unique opportunity to evaluate the niche use of these agents in a highly specialized patient population.

During the study period, a total of 2,119 oncohematology patients were treated at the hospital. Of these, only patients receiving Pt-based therapy were included in the analysis, resulting in a study cohort of 44 patients. The majority of included patients had Hodgkin lymphoma (approximately 40%) or diffuse large B-cell lymphoma (about 43%) (Table S8). Initial treatment regimens did not include Pt-based agents and instead comprised combinations such as doxorubicin, bleomycin, vinblastine, and dacarbazine (ABVD); rituximab, cyclophosphamide, doxorubicin, vincristine, and prednisolone (R-CHOP); and etoposide, prednisone, vincristine, cyclophosphamide, and doxorubicin (EPOCH). Following multiple cycles (around 6 cycles) and subsequent disease progression, all patients were transitioned to a protocol incorporating either cisplatin or carboplatin in combination with other drugs (rituximab, polatuzumab, bortezomib, cyclophosphamide, etoposide, vincristine, vinorelbine etc.). Cisplatin was administered to 14 patients, while 30 received carboplatin. Oxaliplatin was not used in any patients with oncohematology diseases. Only three patients were initially treated with carboplatin, which was later replaced by cisplatin. These patients had diagnoses corresponding to ICD codes C82.7, C83.3, and C84.5.

Hospital-level percentages of oncohematology patients treated with Pt-based drugs in 2024, along with the corresponding number of diagnoses, indicate a low utilization of these therapies. Cisplatin was administered to 14 patients (0.7% of all oncohematology patients) and was used across 4 diagnoses (1.8% of all diagnoses). Carboplatin was administered to 30 patients (1.4%) and was associated with 8 diagnoses (3.7%).

### Current trends in clinical trials with Pt-drugs

A total of 121 ongoing and not recruiting clinical trials (CTs) involving the globally approved Pt-based drugs cisplatin, carboplatin, and oxaliplatin were identified in *ClinicalTrials.gov* for the period 2022–2025, 66 of which included a phase II component (60 phase II, 5 phase II/III, and 1 phase IIa). Among the records retrieved, the most frequently listed study locations were China (*n* = 50) and the United States (*n* = 45). These counts describe the composition of the selected registry dataset and should not be interpreted as measures of comparative national clinical research activity (Table S9).

**Fig. 7:**
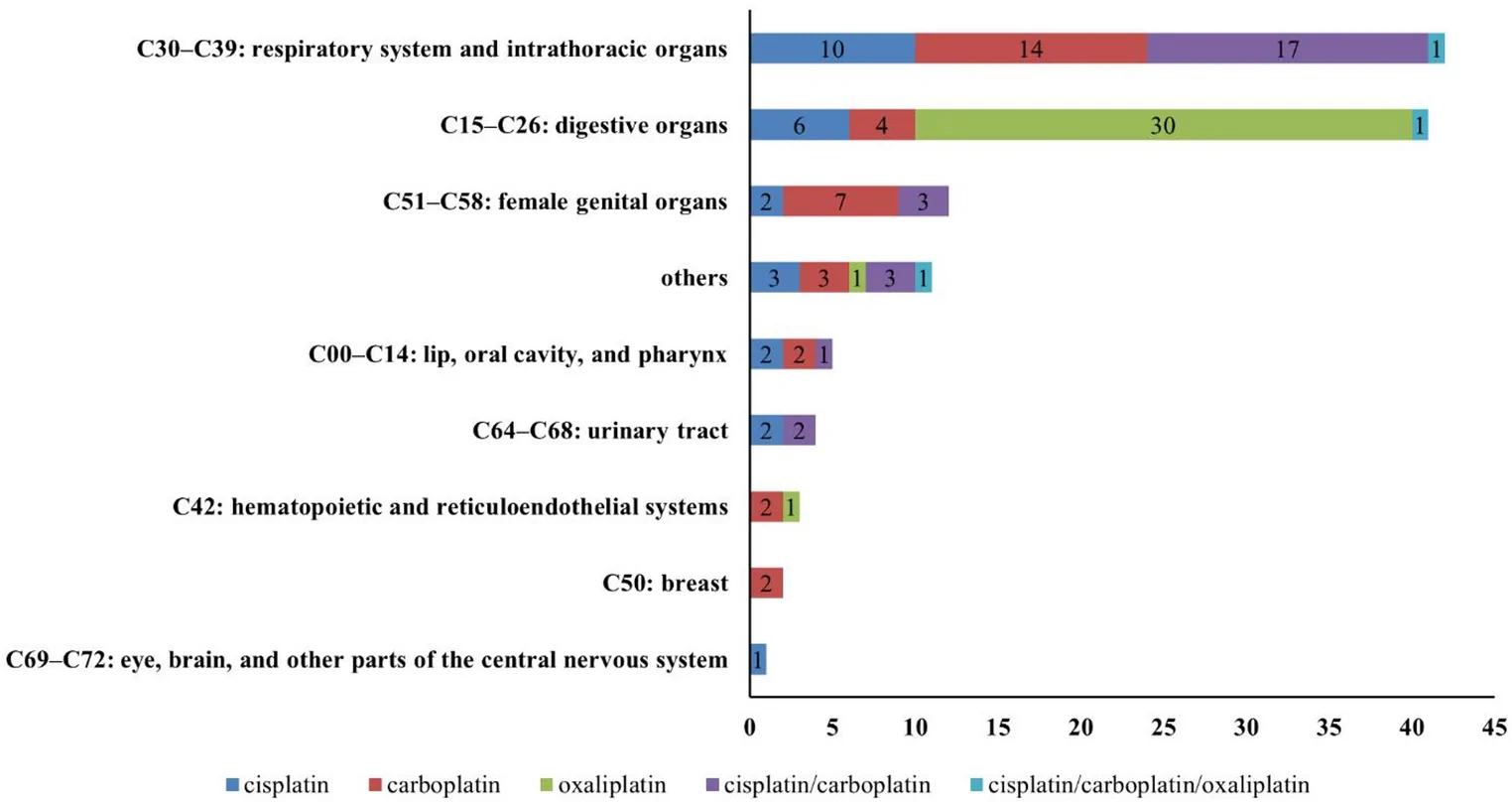
Number of clinical trials with the globally approved Pt-drugs classified by disease category according to ICD-O, identified in ClinicalTrials.gov for the period 2022–2025 The category “Others” includes neoplasms, which are not explicitly specified in the trial (solid tumors; non-haematological cancers such as breast, ovary and head and neck squamous cell carcinomas or not specified at all)

The analysis of tumor types included in the identified CTs reveals a predominance of malignancies with established sensitivity to Pt-based chemotherapy. Non-small cell lung cancer (NSCLC) is the most frequently studied indication (*n* = 22), followed by gastric (*n* = 11), colorectal (*n* = 9), nasopharyngeal (*n* = 8), and small cell lung cancer (*n* = 7). When classified according to anatomical localization using the ICD-O framework, the malignancies included across the identified CTs are mainly distributed within the categories C30–C39 (respiratory and intrathoracic organs) (*n* = 42) and C15–C26 (digestive organs) (*n* = 41), followed by C51-C58 (female genital organs) (*n* = 12) (Fig. 7). This distribution aligns with guideline recommendations and resembles national utilization patterns in Bulgaria, analyzed in the present study, where cancers of the digestive system and lung are among the most prevalent and most frequently treated with Pt-based therapies. The concentration of clinical trials within these same categories suggests that research efforts are focused on optimizing established indications rather than expanding platinum use into entirely new oncological settings.

The most commonly investigated Pt-drugs were carboplatin (*n* = 34), followed by oxaliplatin (*n* = 32) and cisplatin (*n* = 26). Across 26 of the analyzed CTs, the protocol allowed a choice between cisplatin and carboplatin as the platinum agent, without specifying a predefined selection. In three further studies, the protocol allowed selection among multiple platinum agents (cisplatin or oxaliplatin; carboplatin or oxaliplatin; or cisplatin, carboplatin, or oxaliplatin). The trend for carboplatin is consistent with real-world prescribing patterns and guideline recommendations, where it is often preferred over cisplatin in clinical practice due to its more favorable toxicity profile, particularly in patients with impaired renal function or reduced performance status [58]. The use of oxaliplatin in these clinical trials likely reflects the increasing incidence and prevalence of cancers affecting the digestive system, as also demonstrated in the present study.

The majority of clinical trials (68%, *n* = 82) evaluate Pt-based drugs as part of new combination regimens, most commonly with tyrosine kinase inhibitors (TKIs) (*n* = 15), including lenvatinib, brigatinib, anlotinib, futibatinib, fruquintinib, as well as monoclonal antibodies such as pembrolizumab (*n* = 16), tislelizumab (*n* = 9), serplulimab (*n* = 4), and durvalumab (*n* = 4). The investigated therapies are predominantly evaluated in malignancies in which Pt-based chemotherapy remains a standard therapeutic backbone, such as NSCLC and gastric, esophageal, nasopharyngeal, hepatobiliary, cervical, and head and neck malignancies, consistent with the guideline analyses presented in this paper. TKIs were investigated in combination with carboplatin-, cisplatin-, oxaliplatin-, nedaplatin-, or lobaplatin-containing regimens, frequently together with immune checkpoint inhibitors, chemoradiotherapy, hepatic arterial infusion chemotherapy (HAIC), transarterial chemoembolization (TACE), or other multimodal therapeutic approaches (Table S9). The analysis shows that not only modern immunotherapeutic agents and TKIs, but also novel anticancer drug candidates are tested either in combination with Pt-drugs or against established Pt-based therapies in around 30 CTs. The investigated agents target immune modulation pathways, including checkpoint inhibition and bispecific antibody approaches (e.g., QL1706 in NCT05329025 [registration date: 7 April 2022] and ONO-4578 in NCT06256328), as well as targeted signaling inhibition (e.g., DKN-01 in NCT05480306, futibatinib in NCT05945823, and RP-6306 in NCT06107868) and antibody–drug conjugate strategies (e.g., SHR-A1921 in NCT05765032).

In contrast, only a limited proportion of studies (3%) specifically address toxicity mitigation, indicating that despite the well-recognized adverse effects profile of Pt-agents, this aspect remains relatively less explored in current clinical development. The few identified trials in this area primarily investigate supportive or adjunctive strategies (such as N-acetylcysteine, pirenzepine, SGLT2 inhibitors). For instance, trial NCT06019520 evaluates the addition of N-acetylcysteine to cisplatin-based chemotherapy as a potential protective agent [59], while NCT05488873 investigates topical pirenzepine (WST-057) compared to placebo for reducing chemotherapy-induced (carboplatin + paclitaxel) peripheral neuropathy [60]. Similarly, NCT07018622 explores the use of SGLT2 inhibitors in combination with cisplatin or carboplatin [61], and NCT05628233 assesses modifications of cisplatin-based chemotherapy regimens aimed at improving tolerability [62].

Additional clinical trials involving regionally approved platinum agents—nedaplatin (*n* = 26), lobaplatin (*n* = 15, including three trials in which it was permitted as an alternative to nedaplatin), and dicycloplatin (*n* = 1)—were also identified in ClinicalTrials.gov, with approximately half focusing on digestive system cancers, as most were conducted in China (Table S9). This pattern may reflect the high burden of gastrointestinal malignancies, such as gastric and esophageal cancers, in Asian populations [63]. Most trials evaluated these agents as components of multimodal combination regimens, frequently alongside immune checkpoint inhibitors, radiotherapy, or locoregional treatments such as TACE or hyperthermic intraperitoneal chemotherapy (HIPEC), rather than as monotherapy. Nedaplatin was investigated particularly in combination with monoclonal antibodies for esophageal, lung, and head and neck cancers, whereas lobaplatin was evaluated across a broader range of malignancies; the single dicycloplatin trial assessed its use in TACE for hepatocellular carcinoma (Table S9).

Among the investigational Pt-based agents considered, PT-112 was identified in one clinical trial meeting the predefined inclusion criteria. PT-112 (phosplatin) is a first-in-class metallo-pyrophosphate conjugate with a distinct mechanism of action that includes inhibition of ribosome biogenesis and induction of immunogenic cell death [64]. One ongoing phase II, open-label, single-arm study initiated in the United States in 2022 (NCT05104736) is evaluating PT-112 in patients with relapsed or refractory thymic epithelial tumors, including thymoma and thymic carcinoma, for whom therapeutic options after Pt-based chemotherapy are limited [65]. The primary objective is to determine the objective response rate, with study completion expected in 2028. Six additional PT-112 studies registered in ClinicalTrials.gov were initiated before the selected 2022–2025 study period and were therefore outside the scope of the analysis.

Overall, the current landscape of clinical trials involving Pt-based drugs is characterized by an alignment with established clinical guidelines. Rather than expanding platinum use into entirely new indications, most trials have focused on optimizing existing Pt-based treatment regimens by incorporating immunotherapeutic, TKIs, and other novel drug candidates into multimodal treatment strategies, as demonstrated by Yusoh et al. [66]. This reflects the move toward more personalized, combination-based approaches in oncology, while confirming the ongoing importance of platinum agents in cancer therapy.

### Current trends in Pt-based drugs research

#### Growth dynamics of Pt-based drugs research

**Fig. 8:**
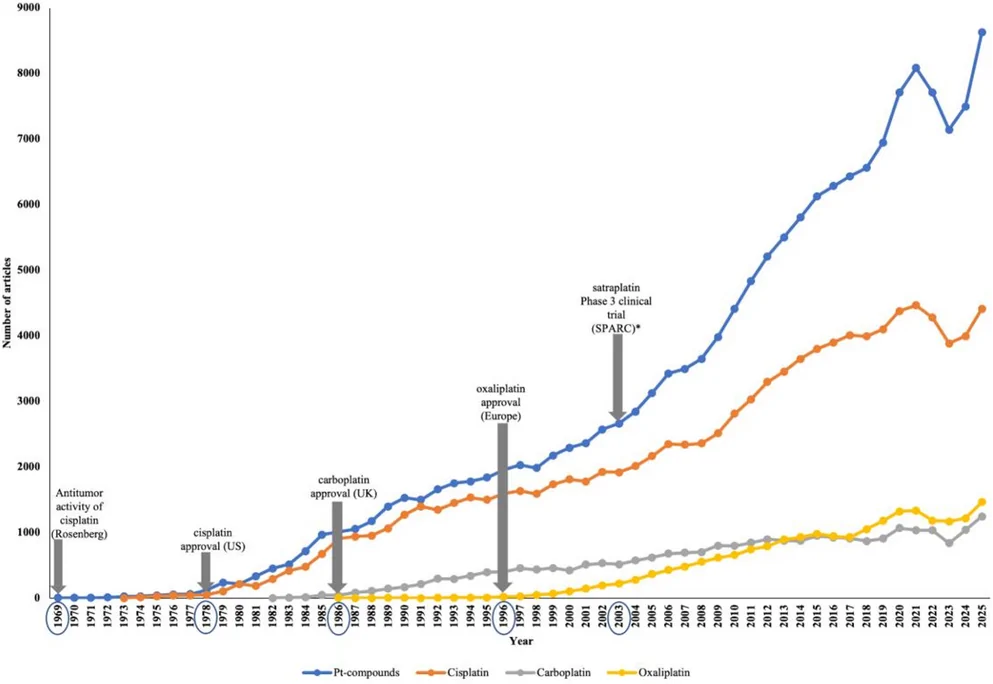
Annual publication trends for Pt-based anticancer compounds (1969–2025), including overall and compound-specific analyses (cisplatin, carboplatin, oxaliplatin). Data source: PubMed (see Methodology). *First orally active Pt(IV) complex (satraplatin) enters phase III clinical trials - Satraplatin and Prednisone Against Refractory Cancer (SPARC) study.

The temporal evolution of publications on Pt-based anticancer compounds indexed in PubMed reveals distinct growth phases (Fig. 8), with trends for the individual clinical agents cisplatin, carboplatin, and oxaliplatin, consistent with those reported by Cheff et al. [67]. Approximately 14% of the identified publications on Pt-based anticancer compounds relate to clinical trials, a proportion comparable to those observed in our compound-specific analyses for cisplatin (13%), carboplatin (22%), and oxaliplatin (15%).

The periods of observation can be divided into four phases: early phase (1969–1978) with relatively small numbers but rapid relative growth following the discovery of antitumor activity of cisplatin [13]; a steady growth (1979–1999) with a gradual increase, including the approval of carboplatin and oxaliplatin; an acceleration (2000–2012) with a clear upward shift and rapid expansion, and a mature growth (2013–2025) with increasing numbers which look more stable. After a period of expansion from 1979 to 1999 (compound annual growth rate, CAGR = 11.8%), the field experienced rapid growth between 2000 and 2012 (CAGR = 7.1%). In the last period (2013–2025), growth is around 3.8%, indicating a mature and still expanding area of research. Overall, the data highlight a shift from steady to accelerated growth around the early 2000s. If, CAGR is calculated for the entire period from 1969 to 2025, the number of publications on Pt-based compounds increased at an average annual rate of 15.3%. This reflects long-term growth and it is influenced by the low number of publications in the early years and does not present the actual growth dynamics.

Key aspects of Pt-based drugs research include a deeper understanding of the medicinal chemistry and pharmacology of platinum chemotherapeutics, the design and development of new Pt-based drugs candidates, the development of new formulations and the exploration of drug repurposing strategies.

#### Development of new Pt-based drugs

The development of new antitumor Pt complexes has evolved over time from classical Pt(II) complexes, following the structure–activity relationships (SAR) based on cisplatin as lead structure [68], toward nonclassical systems, including *trans-*, monofunctional (can bind to DNA through only one coordination site) and noncovalently interacting Pt(II) complexes, multinuclear species, and Pt(IV) compounds [11, 69]. Pt–N-heterocyclic carbene (NHC) complexes and multifunctional Pt(IV) prodrugs represent more recent developments that have gained increasing attention over the last decade. After the first report in 2010 on the cytotoxicity of Pt–NHC complexes and their potential as anticancer drug candidates [70], publications in this area increased at an average rate of around 7.6% per year between 2010 and 2025 (Fig. S3). This class of compounds could offer alternative mechanisms of action, including modes of DNA interaction distinct from those of current Pt drugs and/or the induction of immunogenic cell death, thereby helping to circumvent resistance to Pt-based therapies [71–74]. A couple of recent reviews provide overviews of anticancer Pt–NHC complexes [75, 76].

Multifunctional or multi-action Pt(IV) prodrugs refer to compounds that, upon reduction inside cancer cells, release at least one bioactive ligand in addition to the cytotoxic Pt(II) species [77]. They typically combine the equatorial ligand sphere of a classical anticancer Pt(II) drug with one or two biologically active and/or tumor-targeting moieties attached at the axial positions to overcome resistance and/or increase selectivity toward cancer cells [78, 79]. Early examples include the cisplatin-based Pt(IV) compounds etachraplatin and mitaplatin, developed in the groups of P. Dyson and S. Lippard, respectively [80, 81]. Interest in this field has grown significantly over the last decade (Fig. S3), with publication output increasing by approximately 22.5% per year, and several reviews have comprehensively summarized the recent advances and current status of multifunctional Pt(IV) prodrugs [77, 78, 82–84].

##### New drug formulations

A brief PubMed search covering the period 2022–2025 identified 39 relevant articles on innovative drug delivery systems and emerging formulations for Pt-based chemotherapeutics, including cisplatin, carboplatin, and oxaliplatin. The investigated systems included lipid-based and liposomal formulations (*n* = 13), polymeric nanoparticles/carriers (*n* = 8), hybrid multifunctional nanocarriers (*n* = 6), hydrogels and thermo-responsive systems (*n* = 5), inorganic nanoparticles (*n* = 5), biological carriers (*n* = 3), and supramolecular systems (*n* = 2). These systems offer benefits such as controlled and sustained drug release [85–87], enhanced tumor targeting [88], reduced systemic toxicity [89], reversal of chemoresistance [90–97], and improved immunogenic response, leading to greater therapeutic efficacy with fewer side effects [87, 98–100]. PEGylated liposomal formulations of oxaliplatin have demonstrated improved safety profiles [101] and hybrid nanocarriers co-delivering cisplatin and small interfering RNA (siRNA) reduce drug resistance and enhance efficacy [90]. Similarly, RBC membrane-coated carboplatin nanoparticles improve targeted delivery and cellular uptake [86], and advanced nanocomplexes or hydrogel-based systems further enhance antitumor activity and immune responses [87, 94]. For a recent comprehensive review on current strategies in drug carriers and delivery systems for Pt-based chemotherapeutics, interested readers are referred to Liu et al. [102].

Investigations on advanced drug delivery systems were reported not only for clinically used Pt agents, but also for experimental Pt complexes. In this context, a recent example from our research group demonstrated the potential of bacterial ghost cells derived from nonpathogenic *E. coli* strains as a carrier system for the delivery of Pt–NHC complexes to cancer cells [103].

Extending beyond noncovalent encapsulation of Pt drugs and drug candidates, nanodelivery of Pt(IV) prodrugs can also be achieved through covalent attachment of nanocarriers, such as inorganic or polymeric nanoparticles, metal–organic frameworks, carbon nanotubes, and polymer micelles, to one or both axial ligands [69, 104–106].

#### Repurposing strategies involving anticancer Pt-based drugs

A focused PubMed search on repurposing strategies involving anticancer Pt-based drugs identified 48 relevant studies published between 2022 and 2025, covering applications across oncology and other disease areas. The included literature could be broadly categorized into three main themes: (i) repurposing approved drugs to reduce treatment-related toxicities associated with Pt-based therapy (*n* = 8), (ii) repurposing approved drugs to overcome platinum resistance or enhance the efficacy of Pt-based therapies (*n* = 29), and (iii) repurposing platinum drugs (alone or in combinations) for alternative indications (*n* = 11).

Studies focusing on platinum resistance and efficacy enhancement identified synergistic or potentiating effects with cisplatin, carboplatin, or oxaliplatin for several approved drug classes, including statins (pitavastatin) [107, 108], antipsychotics (aripiprazole, pimozide, penfluridol) [109, 110], antifungals (oxiconazole) [111], beta-blockers (propranolol) [112], selective estrogen receptor modulators (bazedoxifene) [113], antifibrotic agents (pirfenidone) [114], potassium-sparing diuretics (spironolactone, triamterene) [115, 116], gold-containing anti-rheumatic agents (auranofin) [117], antiprogestins (mifepristone) [118], antiviral agents (ritonavir) [119], anesthetic agents (propofol) [120], antidiabetic drugs (metformin) [107], aldehyde dehydrogenase inhibitors (disulfiram) [121], macrocyclic immunosuppressive drugs (rapamycin) [122], and bioactive natural products (derived from *Astragalus membranaceus*, *Angelica gigas*, *Trichosanthes kirilowii*, and formononetin) [123]. Earlier work from 2019 further demonstrated that systematic screening of drug libraries represents a promising strategy for identifying compounds that potentiate the activity or synergize with cisplatin, carboplatin, or oxaliplatin, particularly in chemoresistant cancers [124].

In parallel, several studies investigated repurposed agents aimed at mitigating major platinum-associated toxicities, including nephroprotective strategies against cisplatin-induced kidney injury or nephrotoxicity (dabrafenib [125], diphenhydramine [126], papaverine [127], and 4-methylpyrazole [128]), approaches to reduce the overall toxicity of cisplatin (dihydroartemisinin [129]), and interventions targeting oxaliplatin-induced peripheral neuropathy (simvastatin [130] and proton pump inhibitors [131]).

A smaller subset of studies (*n* = 11/48) explored the repurposing potential of Pt-drugs across different cancer types, including advanced immune checkpoint blockade (ICB)-resistant melanoma [132], gastric cancer (cisplatin plus procaterol) [133], chordoma (cisplatin), glioblastoma [134], and lung squamous cell carcinoma (oxaliplatin plus afatinib). Additional studies investigated non-oncological applications, including the rare disease amyotrophic lateral sclerosis (azacitidine plus carboplatin) [135], SARS-CoV-2 infection (cisplatin and carboplatin) [136, 137], type 2 diabetes mellitus (cisplatin) [138], and bacterial diseases such as *H. pylori* infection [139]. Overall, evidence was predominantly preclinical, derived from in silico, in vitro, xenograft or organoid models, highlighting promising but early-stage repurposing opportunities that require further clinical validation. It is noteworthy that new platinum drug candidates are also being investigated beyond oncology, particularly as antimicrobial agents against multidrug-resistant pathogens [140–142]. In this context, multifunctional Pt(IV) complexes featuring axial deferoxamine-based ligands have recently emerged as promising anti-infective theranostic agents for life-threatening infections [143].

These repurposing efforts may be particularly relevant in the context of rare cancers, which account for approximately 25% of all cancer cases and are often associated with limited therapeutic options due to sparse clinical evidence and a lack of approved targeted treatments. Pt-based chemotherapeutics, owing to their broad cytotoxic activity, therefore represent attractive candidates for repurposing strategies aimed at addressing these unmet needs through resistance modulation, improved delivery, and treatment individualization.

## Conclusion

In conclusion, Pt-based drugs continue to play a central role in clinical oncology across a wide range of malignancies. Analysis of national and international guideline sources showed that Pt-containing treatment regimens were recommended in more than half (56%–83%) of the 18 ICD-O topographic categories, demonstrating their broad and continuing clinical relevance. Real-world data from the Bulgarian case study further support their sustained and, in some indications, increasing clinical use, particularly in cancers where platinum sensitivity remains high. At the same time, their application is still limited by well-known challenges such as systemic toxicity and the development of resistance. Rather than being replaced by newer therapies, current clinical research also focuses on improving Pt-based treatments through combinations with immunotherapy and targeted agents, while advanced drug delivery approaches are actively being explored.

Taken together, our findings reinforce the view proposed by a previous mini-perspective that Pt-based drugs are and will remain a key component of cancer treatment in the near future [67], while ongoing efforts are aimed at improving their safety and expanding their clinical applications. Moreover, the growing number of publications and the development of new approaches, including multi-action Pt(IV) prodrugs and Pt–NHC compounds, indicate that Pt-based therapeutics remain an active and evolving field of research, potentially paving the way for the next breakthrough Pt-drug.

### Author contributions

M.K. conducted the formal analysis and investigation, prepared the original draft, and provided resources. H.V. conceptualized and supervised the study, contributed to the methodology, and reviewed and edited the manuscript. M.K. and H.V. developed the methodology. All authors reviewed the manuscript.

### Funding

No funding source is available for the current project.

## Supplementary Information

Below is the link to the electronic supplementary material.

Supplementary Material 1

## Data Availability

Data supporting the findings of the current study are available in the Supplementary file. Should any data be needed in another format they are available from the corresponding authors upon reasonable request.
