# Supplementary material for: Current clinical applications and future developments of platinum-based anticancer drugs
Source: J Biol Inorg Chem. 2026 Sep 10;31(6):469–88. doi: 10.1007/s00775-026-02172-x (PMC13645941; doi:10.1007/s00775-026-02172-x)
Supplement: Supplementary file 1 [file 775_2026_2172_MOESM1_ESM.docx]

**Current clinical applications and future developments of platinum-based anticancer drugs**

Maria Kamusheva^1^, Hristo P. Varbanov^2,3^

*^1^Faculty of Pharmacy, Medical University of Sofia, Sofia, Bulgaria*

*^2^Institute of Pharmacy-Pharmaceutical Chemistry,University of Innsbruck, Innsbruck, Austria*

*^3^ Medical College, Trakia University-Stara Zagora, Stara Zagora, Bulgaria*

**Corresponding author:** [mkamusheva@pharmfac.mu-sofia.bg](mailto:mkamusheva@pharmfac.mu-sofia.bg); [hristo.varbanov@uibk.ac.at](mailto:hristo.varbanov@uibk.ac.at)

**Journal Name:** JBIC Journal of Biological Inorganic Chemistry

Supporting Information

**Table of Contents**

[**Literature search strategy**	2](#_Toc238226992)

[**ESMO guidelines**	3](#_Toc238226993)

[**NCCN guidelines**	4](#_Toc238226994)

[**Bulgarian guidelines**	6](#_Toc238226995)

[**Austrian guidelines**	7](#_Toc238226996)

[**Comparative analysis of the guidelines**	9](#_Toc238226997)

[**Utilization data of Pt-based drugs in Bulgaria**	10](#_Toc238226998)

[**Hospital-based analysis**	11](#_Toc238226999)

[**Clinical trials data**	12](#_Toc238227000)

[**Drug utilization data**	41](#_Toc238227001)

[**Neoplasms treated with Pt-drugs**	42](#_Toc238227002)

[**Pt-NHC and multifunctional Pt(IV) complexes papers**	43](#_Toc238227003)

## **Literature search strategy**

**Table S1.** Terms used for the literature search

| Repurposing topic | ((cisplatin OR oxaliplatin OR carboplatin OR "platinum-based drugs")) AND (repurposing OR repositioning OR "drug repurposing" OR "drug repositioning" OR "drug rediscovery") |
| --- | --- |
| Drug formulations topic | ((cisplatin OR carboplatin OR oxaliplatin OR "platinum-based drugs")) AND ("drug delivery systems" OR "drug formulation" OR nanoparticles OR liposomes) |
| Pt-NHC | **("N-heterocyclic carbene*"[Title/Abstract]) AND ( platinum[Title/Abstract] OR "Platinum Compounds"[MeSH Terms] ) AND ( "Neoplasms"[MeSH Terms] OR cancer*[Title/Abstract] OR tumor*[Title/Abstract] OR tumour*[Title/Abstract] OR neoplas*[Title/Abstract] OR cytotoxic*[Title/Abstract])** |
| Pt(IV) multifunctional | ("Pt(IV)"[Title/Abstract] OR "platinum(IV)"[Title/Abstract] ) AND ("Neoplasms"[MeSH Terms] OR cancer*[Title/Abstract] OR tumor*[Title/Abstract] OR tumour*[Title/Abstract] OR neoplas*[Title/Abstract] OR cytotoxic*[Title/Abstract]) AND (multitarget*[Title/Abstract] OR "multi-action"[Title/Abstract] OR "dual-action"[Title/Abstract] OR "triple-action"[Title/Abstract] OR multifunctional[Title/Abstract] OR bifunctional[Title/Abstract] OR dual-threat[Title/Abstract] OR multiaction[Title/Abstract]) |
| Pt-drugs | **((((((((platinum compounds[MeSH Terms]) OR (Platinum/therapeutic use[Mesh Terms]) OR (platinum[Title/Abstract])) OR (Pt(IV[Title/Abstract]))) OR (Pt(II[Title/Abstract]))) AND (((((((neoplasm[MeSH Terms]) OR (cancer[Title/Abstract])) OR (tumour[Title/Abstract])) OR (tumor[Title/Abstract])) OR (cytotoxic[Title/Abstract])) OR (antineoplastic[Title/Abstract])) OR (cytotoxicity[Title/Abstract]))) OR ((carboplatin[MeSH Terms]) OR (carboplatin[Title/Abstract]))) OR ((oxaliplatin[MeSH Terms]) OR (oxaliplatin[Title/Abstract]))) OR ((cisplatin[MeSH Terms]) OR (cisplatin[Title/Abstract])))** |

## **ESMO guidelines**

**Table S2.** Application of Pt-based therapies according to the ESMO guidelines

| Cancer types | Total number of documents (related diseases in the group) | Number of documents (related diseases in the group) containing Pt-based drugs regimens |
| --- | --- | --- |
| Breast cancer | 4 | 3 |
| Cancer in unknown primary | 1 | 1 |
| Endocrine and Neuroendocrine Cancers | 6 | 6 |
| Gastrointestinal Cancers | 10 | 8 |
| Genitorinary | 5 | 4 |
| Gynaecological Cancers | 6 | 6 |
| Haematological Malignancies | 24 | 8 |
| Head and neck cancer | 4 | 4 |
| Hereditary Syndromes | 2 | 0 |
| Lung and Chest Tumours | 9 | 9 |
| Melanoma and skin cancer | 2 | 2 |
| Neuro-Oncology | 4 | 1 |
| Sarcoma and GIST | 3 | 1 |

## **NCCN guidelines**

**Table S3.** Application of Pt-based drugs in treatment regimens according to the NCCN guidelines

| Cancer type | W/o Pt-based drug | With Pt-based drugs |
| --- | --- | --- |
| [Acute Lymphoblastic Leukaemia](https://www.nccn.org/guidelines/guidelines-detail?category=1&id=1410) | **+** |  |
| [Acute Myeloid Leukaemia](https://www.nccn.org/guidelines/guidelines-detail?category=1&id=1411) | **+** |  |
| Ampullary Adenocarcinoma |  | **+** |
| [Anal Carcinoma](https://www.nccn.org/guidelines/guidelines-detail?category=1&id=1414) |  | **+** |
| [Basal Cell Skin Cancer](https://www.nccn.org/guidelines/guidelines-detail?category=1&id=1416) | **+** |  |
| [B-Cell Lymphomas](https://www.nccn.org/guidelines/guidelines-detail?category=1&id=1480) |  | **+** |
| [Biliary Tract Cancers](https://www.nccn.org/guidelines/guidelines-detail?category=1&id=1517) |  | **+** |
| [Bladder Cancer](https://www.nccn.org/guidelines/guidelines-detail?category=1&id=1417) |  | **+** |
| [Bone Cancer](https://www.nccn.org/guidelines/guidelines-detail?category=1&id=1418) | **+** |  |
| [Breast Cancer](https://www.nccn.org/guidelines/guidelines-detail?category=1&id=1419) |  | **+** |
| [Castleman Disease](https://www.nccn.org/guidelines/guidelines-detail?category=1&id=1518) | **+** |  |
| [Central Nervous System Cancers](https://www.nccn.org/guidelines/guidelines-detail?category=1&id=1425) |  | **+** |
| [Cervical Cancer](https://www.nccn.org/guidelines/guidelines-detail?category=1&id=1426) |  | **+** |
| [Chronic Lymphocytic Leukaemia/Small Lymphocytic Lymphoma](https://www.nccn.org/guidelines/guidelines-detail?category=1&id=1478) |  | **+** |
| [Chronic Myeloid Leukaemia](https://www.nccn.org/guidelines/guidelines-detail?category=1&id=1427) | **+** |  |
| [Colon Cancer](https://www.nccn.org/guidelines/guidelines-detail?category=1&id=1428) |  | **+** |
| [Dermatofibrosarcoma Protuberans](https://www.nccn.org/guidelines/guidelines-detail?category=1&id=1430) | **+** |  |
| [Oesophageal and Esophagogastric Junction Cancers](https://www.nccn.org/guidelines/guidelines-detail?category=1&id=1433) | **+** |  |
| [Gastric Cancer](https://www.nccn.org/guidelines/guidelines-detail?category=1&id=1434) |  | **+** |
| [Gastrointestinal Stromal Tumours](https://www.nccn.org/guidelines/guidelines-detail?category=1&id=1507) | **+** |  |
| [Gestational Trophoblastic Neoplasia](https://www.nccn.org/guidelines/guidelines-detail?category=1&id=1489) |  | **+** |
| [Hairy Cell Leukaemia](https://www.nccn.org/guidelines/guidelines-detail?category=1&id=1481) | **+** |  |
| [Head and Neck Cancers](https://www.nccn.org/guidelines/guidelines-detail?category=1&id=1437) |  | **+** |
| [Hepatobiliary Cancers](https://www.nccn.org/guidelines/guidelines-detail?category=1&id=1438) | **+** |  |
| [Hepatocellular Carcinoma](https://www.nccn.org/guidelines/guidelines-detail?category=1&id=1514) |  | **+** |
| [Histiocytic Neoplasms](https://www.nccn.org/guidelines/guidelines-detail?category=1&id=1502) | **+** |  |
| [Hodgkin Lymphoma](https://www.nccn.org/guidelines/guidelines-detail?category=1&id=1439) |  | **+** |
| [Kaposi Sarcoma](https://www.nccn.org/guidelines/guidelines-detail?category=1&id=1485) | **+** |  |
| [Kidney Cancer](https://www.nccn.org/guidelines/guidelines-detail?category=1&id=1440) |  | **+** |
| [Melanoma: Cutaneous](https://www.nccn.org/guidelines/guidelines-detail?category=1&id=1492) |  | **+** |
| [Melanoma: Uveal](https://www.nccn.org/guidelines/guidelines-detail?category=1&id=1488) |  | **+** |
| [Merkel Cell Carcinoma](https://www.nccn.org/guidelines/guidelines-detail?category=1&id=1444) |  | **+** |
| [Mesothelioma: Peritoneal](https://www.nccn.org/guidelines/guidelines-detail?category=1&id=1513) |  | **+** |
| [Mesothelioma: Pleural](https://www.nccn.org/guidelines/guidelines-detail?category=1&id=1512) |  | **+** |
| [Multiple Myeloma](https://www.nccn.org/guidelines/guidelines-detail?category=1&id=1445) |  | **+** |
| [Myelodysplastic Syndromes](https://www.nccn.org/guidelines/guidelines-detail?category=1&id=1446) | **+** |  |
| [Myeloid/Lymphoid Neoplasms with Eosinophilia and Tyrosine Kinase Gene Fusions](https://www.nccn.org/guidelines/guidelines-detail?category=1&id=1505) | **+** |  |
| [Myeloproliferative Neoplasms](https://www.nccn.org/guidelines/guidelines-detail?category=1&id=1477) | **+** |  |
| [Neuroblastoma](https://www.nccn.org/guidelines/guidelines-detail?category=1&id=1520) |  | **+** |
| [Neuroendocrine and Adrenal Tumors](https://www.nccn.org/guidelines/guidelines-detail?category=1&id=1448) |  | **+** |
| [Non-Small Cell Lung Cancer](https://www.nccn.org/guidelines/guidelines-detail?category=1&id=1450) |  | **+** |
| [Occult Primary](https://www.nccn.org/guidelines/guidelines-detail?category=1&id=1451) |  | **+** |
| [Ovarian Cancer/Fallopian Tube Cancer/Primary Peritoneal Cancer](https://www.nccn.org/guidelines/guidelines-detail?category=1&id=1453) |  | **+** |
| [Ovarian Cancer/Fallopian Tube Cancer/Primary Peritoneal Cancer](https://www.nccn.org/guidelines/guidelines-detail?category=1&id=1453) |  | **+** |
| [Pediatric Acute Lymphoblastic Leukemia](https://www.nccn.org/guidelines/guidelines-detail?category=1&id=1496) | **+** |  |
| [Pediatric Aggressive Mature B-Cell Lymphomas](https://www.nccn.org/guidelines/guidelines-detail?category=1&id=1504) |  | **+** |
| [Pediatric Central Nervous System Cancers](https://www.nccn.org/guidelines/guidelines-detail?category=1&id=1509) |  | **+** |
| [Pediatric Hodgkin Lymphoma](https://www.nccn.org/guidelines/guidelines-detail?category=1&id=1498) |  | **+** |
| [Penile Cancer](https://www.nccn.org/guidelines/guidelines-detail?category=1&id=1456) |  | **+** |
| [Primary Cutaneous Lymphomas](https://www.nccn.org/guidelines/guidelines-detail?category=1&id=1491) |  | **+** |
| [Prostate Cancer](https://www.nccn.org/guidelines/guidelines-detail?category=1&id=1459) |  | **+** |
| [Rectal Cancer](https://www.nccn.org/guidelines/guidelines-detail?category=1&id=1461) |  | **+** |
| [Small Bowel Adenocarcinoma](https://www.nccn.org/guidelines/guidelines-detail?category=1&id=1495) |  | **+** |
| [Small Cell Lung Cancer](https://www.nccn.org/guidelines/guidelines-detail?category=1&id=1462) |  | **+** |
| [Soft Tissue Sarcoma](https://www.nccn.org/guidelines/guidelines-detail?category=1&id=1464) |  | **+** |
| [Squamous Cell Skin Cancer](https://www.nccn.org/guidelines/guidelines-detail?category=1&id=1465) |  | **+** |
| [Systemic Light Chain Amyloidosis](https://www.nccn.org/guidelines/guidelines-detail?category=1&id=1467) | **+** |  |
| [Systemic Mastocytosis](https://www.nccn.org/guidelines/guidelines-detail?category=1&id=1490) | **+** |  |
| [T-Cell Lymphomas](https://www.nccn.org/guidelines/guidelines-detail?category=1&id=1483) |  | **+** |
| [Testicular Cancer](https://www.nccn.org/guidelines/guidelines-detail?category=1&id=1468) |  | **+** |
| [Thymomas and Thymic Carcinomas](https://www.nccn.org/guidelines/guidelines-detail?category=1&id=1469) |  | **+** |
| [Thyroid Carcinoma](https://www.nccn.org/guidelines/guidelines-detail?category=1&id=1470) |  | **+** |
| [Uterine Neoplasms](https://www.nccn.org/guidelines/guidelines-detail?category=1&id=1473) |  | **+** |
| [Vaginal Cancer](https://www.nccn.org/guidelines/guidelines-detail?category=1&id=1521) |  | **+** |
| [Vulvar Cancer](https://www.nccn.org/guidelines/guidelines-detail?category=1&id=1476) |  | **+** |
| [Waldenström Macroglobulinemia/Lymphoplasmacytic Lymphoma](https://www.nccn.org/guidelines/guidelines-detail?category=1&id=1475) | **+** |  |
| [Wilms Tumour (Nephroblastoma)](https://www.nccn.org/guidelines/guidelines-detail?category=1&id=1500) |  | **+** |

## **Bulgarian guidelines**

**Table S4.** Application of Pt-based drugs according to the Bulgarian guidelines

| Cancer type | Pt -based drug | | |
| --- | --- | --- | --- |
| Primary Brain Tumors |  |  |  |
| Metastatic Brain Tumors |  |  |  |
| Head and Neck Carcinomas | | | |
| *Nasopharyngeal Carcinomas* | cisplatin |  |  |
| *Squamous Cell Carcinomas of the Head and Neck* | cisplatin | carboplatin |  |
| Thyroid Carcinoma | cisplatin |  |  |
| Breast Carcinoma | cisplatin | carboplatin |  |
| Malignant Thymoma and Thymic Carcinoma | cisplatin | carboplatin |  |
| Lung Carcinoma | cisplatin | carboplatin |  |
| Malignant Pleural Mesothelioma | cisplatin |  |  |
| Esophageal Carcinoma | cisplatin | carboplatin |  |
| Stomach Carcinoma | cisplatin |  | oxaliplatin |
| Pancreatic Adenocarcinoma | cisplatin |  | oxaliplatin |
| Hepatocellular Carcinoma | cisplatin |  | oxaliplatin |
| Biliary Carcinoma | cisplatin |  | oxaliplatin |
| Kidney Carcinoma |  |  |  |
| Colorectal Carcinoma |  |  | oxaliplatin |
| Anal Carcinoma | cisplatin |  |  |
| Neuroendocrine Tumors | cisplatin | carboplatin |  |
| Carcinoma of the Ovary, Fallopian Tube, and Primary Peritoneal Carcinoma | cisplatin | carboplatin |  |
| Endometrial Carcinoma | cisplatin | carboplatin |  |
| Trophoblastic Disease | cisplatin |  |  |
| Cervical Carcinoma | cisplatin | carboplatin |  |
| Malignant Vaginal Tumors | cisplatin |  |  |
| Malignant Vulvar Tumors | cisplatin |  |  |
| Bladder Carcinoma | cisplatin | carboplatin |  |
| Prostate Carcinoma | cisplatin |  |  |
| Testicular Carcinoma | cisplatin | carboplatin |  |
| Penile Carcinoma | cisplatin |  |  |
| Kaposi’s Sarcoma |  |  |  |
| Soft Tissue Sarcomas | cisplatin |  |  |
| Osteosarcomas | cisplatin | carboplatin |  |
| Giant Cell Tumor of Bone |  |  |  |
| Gastrointestinal Stromal Tumors (GISTs) |  |  |  |
| Malignant Melanoma | cisplatin | carboplatin |  |
| Skin Carcinoma |  |  |  |
| Carcinoma of Unknown Primary (CUP) | cisplatin | carboplatin | oxaliplatin |
| Lymphoid Neoplasms | | | |
| *Diffuse Large B-Cell Lymphoma* |  |  | oxaliplatin |
| *Lymphomas, Chronic Lymphocytic Leukemia (CLL)* | cisplatin | carboplatin | oxaliplatin |
| *Hodgkin Lymphomas* |  | carboplatin |  |
| *Plasma Cell Neoplasms* | cisplatin |  |  |
| Benign Hematologic Diseases |  |  |  |
| Myeloid Neoplasms |  |  |  |
| Lymphoid Neoplasms |  |  |  |
| Hematopoietic Stem Cell Transplantation |  |  |  |

## **Austrian guidelines**

**Table S5**. Pt-based treatment regimens listed in the Austrian guidelines

| Pt-drug | In combination | Disease | Regimen |
| --- | --- | --- | --- |
| *carboplatin* |  | ovarian cancer | chemotherapy-based |
| *carboplatin* | cyclophosphamide | ovarian cancer | chemotherapy-based |
| *carboplatin* | docetaxel | ovarian cancer | chemotherapy-based |
| *carboplatin* | atezolizumab+bevacizumab+paclitaxel | non-small cell lung cancer | antibody-based |
| *carboplatin* | atezolizumab+etoposide | small cell lung cancer | antibody-based |
| *carboplatin* | atezolizumab+nab-paclitaxel | non-small cell lung cancer | antibody-based |
| *carboplatin* | paclitaxel+bavacizumab | ovarian cancer | antibody-based |
| *carboplatin* | durvalumab+etoposide | small cell lung cancer | antibody-based |
| *carboplatin* | pembrolizumab+gemcitabine | breast cancer | antibody-based |
| *carboplatin* | pembrolizumab+paclitaxel | cervical and non-small cell lung cancer | antibody-based |
| *carboplatin* | pembrolizumab+pemetrexed | non-small cell lung cancer | antibody-based |
| *carboplatin* | pembrolizumab+paclitaxel | breast cancer | antibody-based |
| *cisplatin* | raltitrexed | mesothelioma | chemotherapy-based |
| *cisplatin* | cytarabine+dexamethasone | high grade NHL | chemotherapy-based |
| *cisplatin* | etoposide | neuroendocrine tumor | chemotherapy-based |
| *cisplatin* | etoposide | small cell lung cancer | chemotherapy-based |
| *cisplatin* | gemcitabine | breast cancer | chemotherapy-based |
| *cisplatin* | gemcitabine | urothelial carcinoma | chemotherapy-based |
| *cisplatin* | etoposide+bleomycin | testicular cancer | chemotherapy-based |
| *cisplatin* | etoposide+ifosfamide | testicular cancer | chemotherapy-based |
| *cisplatin* | cetuximab+5-FU | head and neck cancer | antibody-based |
| *cisplatin* | docetaxel+cetuximab | head and neck cancer | antibody-based |
| *cisplatin* | durvalumab+gemcitabin | cholangiocellular carcinoma | antibody-based |
| *cisplatin* | durvalumab+pemetrexed | mesothelioma | antibody-based |
| *cisplatin* | nivolumab+5-FU | esophageal cancer | antibody-based |
| *cisplatin* | 5-FU+pembrolizumab | esophageal cancer | antibody-based |
| *cisplatin* | pembrolizumab+5-FU | head and neck cancer | antibody-based |
| *cisplatin* | capecitabine+trastuzumab | gastric cancer | antibody-based |
| *oxaliplatin* | capecitabine | colorectal cancer | chemotherapy-based |
| *oxaliplatin* | irinotecan+calcium folinate+5-FU | pancreatic cancer | chemotherapy-based |
| *oxaliplatin* | Ca folinate+5-FU | gastric cancer | chemotherapy-based |
| *oxaliplatin* | Ca folinate+5-FU+docetaxel | gastric cancer | chemotherapy-based |
| *oxaliplatin* | irinotecan | gastric cancer | chemotherapy-based |
| *oxaliplatin* | Ca folinate+5-FU | pancreatic cancer | chemotherapy-based |
| *oxaliplatin* | Ca-folinate+5-FU+bevacizumab | colorectal cancer | antibody-based |
| *oxaliplatin* | bevacizumab+irinotecan+Ca-folinate+5-FU | colorectal cancer | antibody-based |
| *oxaliplatin* | cetuximab+Ca-folinate+5-FU | colorectal cancer | antibody-based |
| *oxaliplatin* | nivolumab+Ca-folinate+5-FU | gastric cancer | antibody-based |
| *oxaliplatin* | panitumumab+Ca-folinate+5-FU | colorectal cancer | antibody-based |
| *oxaliplatin* | rituximab+gemcitabine | high grade NHL | antibody-based |

## **Comparative analysis of the guidelines**

**Table S6.** Presence of treatment protocols containing Pt-based drugs by ICD-O topography categories across the analyzed guidelines

| ICD-O classification by topography criteria | NCCN | ESMO | Bulgarian | Austrian | Australian |
| --- | --- | --- | --- | --- | --- |
| *C00-C14 LIP, ORAL CAVITY AND PHARYNX* | + | + | + | + | + |
| *C15-C26 DIGESTIVE ORGANS* | + | + | + | + | + |
| *C30-C39 RESPIRATORY SYSTEM AND INTRATORACIC ORGANS* | + | + | + | + | + |
| *C40-C41 BONES, JOINTS and ARTICULAR CARTILAGE* |  |  | + |  | + |
| *C42 HEMATOPOIETIC AND RETICULOENDOTHELIAL SYSTEMS* | + | + | + | + | + |
| *C44 SKIN* | + | + |  |  | + |
| *C47 PERIPHERAL NERVES AND AUTONOMIC NERVOUS SYSTEM* | + | + |  |  | + |
| *C48 RETROPERITONEUM AND PERITONEUM* | + |  |  |  |  |
| *C49 CONNECTIVE, SUBCUTANEOUS AND OTHER SOFT TISSUES* | + | + | + | + | + |
| *C50 BREASТ* | + | + | + | + | + |
| *C51-C58 FEMALE GENITAL ORGANS* | + | + | + | + | + |
| *C60-C63 MALE GENITAL ORGANS* | + |  | + | + | + |
| *C64-C68 URINARY TRACT* | + | + | + | + | + |
| *C69-C72 EYE, BRAIN AND OTHER PARTS OF CENTRAL NERVOUS SYSTEM* | + |  |  |  |  |
| *C73-C75 THYROID AND OTHER ENDOCRINE GLANDS* | + | + | + | + |  |
| *С76 OTHER AND ILL-DEFINED SITES* |  |  |  |  |  |
| *C77 LYMPH NODES* |  |  |  |  |  |
| *C80 UNKNOWN PRIMARY SITE* | + | + | + |  |  |
| *%* | 83.3% | 66.7% | 72.2% | 55.6% | 66.7% |

## **Utilization data of Pt-based drugs in Bulgaria**

**Table S7.** Number of patients treated with Pt-based drugs by diagnosis according to the ICD for the period 2021-2024 in Bulgaria

|  | **01.01.2021** | | | **01.12.2021** | | | **01.01.2022** | | | **01.12.2022** | | | **01.01.2023** | | | **01.12.2023** | | | **01.01.2024** | | | **01.12.2024** | | |
| --- | --- | --- | --- | --- | --- | --- | --- | --- | --- | --- | --- | --- | --- | --- | --- | --- | --- | --- | --- | --- | --- | --- | --- | --- |
|  | **Cis** | **Ca** | **Oxa** | **Cis** | **Ca** | **Oxa** | **Cis** | **Ca** | **Oxa** | **Cis** | **Ca** | **Oxa** | **Cis** | **Ca** | **Oxa** | **Cis** | **Ca** | **Oxa** | **Cis** | **Ca** | **Oxa** | **Cis** | **Ca** | **Oxa** |
| C00-C14 | 69 | 17 |  | 77 | 17 |  | 77 | 18 |  | 95 | 13 |  | 83 | 13 |  | 67 | 34 |  | 59 | 50 |  | 78 | 30 |  |
| C15-C26 | 93 | 23 | 952 | 101 | 20 | 1109 | 76 | 16 | 1125 | 92 | 17 | 1153 | 132 | 21 | 1233 | 101 | 46 | 1365 | 93 | 61 | 1557 | 120 | 43 | 1334 |
| C30-C39 | 113 | 284 |  | 143 | 321 | 2 | 37 | 54 | 2 | 36 | 60 |  | 123 | 365 |  | 105 | 381 | 1 | 88 | 443 |  | 121 | 402 | 1 |
| C40-C41 | 1 |  |  |  | 1 |  | 1 | 1 |  |  |  |  | 1 |  |  |  | 1 |  |  |  |  |  |  |  |
| C42 | 16 | 4 | 1 |  | 3 | 1 | 16 | 6 |  | 10 | 7 | 1 | 19 | 6 | 1 | 13 | 3 | 1 | 13 | 1 | 5 | 5 | 12 |  |
| C44 |  | 4 |  | 12 | 5 |  | 1 | 3 |  | 4 | 4 |  | 2 | 6 |  |  | 8 |  | 3 | 7 |  | 2 | 4 |  |
| C47 |  |  |  |  |  |  |  |  |  |  |  |  |  |  |  |  |  |  |  | 4 |  |  |  |  |
| C48 |  | 3 |  |  | 7 |  |  | 4 |  | 1 | 2 | 1 | 2 | 4 | 1 |  | 4 |  | 1 |  |  | 1 | 4 | 1 |
| C49 |  | 1 |  | 2 | 1 |  | 2 |  |  | 1 |  |  | 1 | 1 |  | 1 |  |  | 2 |  |  | 1 |  |  |
| C50 | 9 | 58 |  | 20 | 75 | 1 | 22 | 65 | 1 | 12 | 98 | 2 | 17 | 102 | 2 | 7 | 97 |  | 3 | 105 |  | 6 | 151 |  |
| C51-C58 | 91 | 396 | 1 | 85 | 407 |  | 22 | 55 |  | 25 | 57 |  | 99 | 446 |  | 75 | 489 | 2 | 61 | 508 | 2 | 82 | 404 |  |
| C60-C63 | 31 | 9 |  | 40 | 9 |  | 45 | 10 |  | 28 | 11 |  | 37 | 9 |  | 22 | 7 |  | 21 | 13 | 1 | 37 | 10 |  |
| C64-C68 | 33 | 28 | 1 | 35 | 23 | 1 | 30 | 19 |  | 42 | 43 |  | 52 | 38 |  | 38 | 51 |  | 26 | 72 |  | 39 | 41 | 1 |
| C69-C72 | 1 | 3 |  |  |  |  |  |  |  |  |  |  | 2 |  |  | 1 | 1 |  | 2 | 1 |  | 1 |  |  |
| C73-C75 |  |  |  |  |  |  |  | 1 |  | 1 |  |  | 2 |  |  | 4 | 1 |  |  | 1 |  | 1 | 1 |  |
| С76 | 4 | 7 |  | 1 | 6 | 1 | 3 | 2 |  | 5 | 4 | 1 | 6 | 9 |  | 3 | 4 |  | 5 | 4 |  | 4 | 7 |  |
| C77 | 12 | 10 | 10 | 3 | 6 | 7 | 5 | 7 | 9 | 3 |  |  | 1 | 1 |  | 5 |  |  |  |  |  |  |  |  |
| C80 by site | 4 | 6 | 3 | 2 | 6 |  | 4 | 4 | 2 | 2 | 3 | 2 | 4 | 10 | 1 |  | 9 | 3 | 6 | 9 | 2 | 10 | 10 | 3 |

*Cis - cisplatin; Ca - carboplatin; Oxa – oxaliplatin; ICD – International Classification of Diseases*

## **Hospital-based analysis**

**Table S8.** Characteristics of the patients treated with Pt-drugs at the hospital of interest (2024)

| Patients characteristics | Cisplatin | Carboplatin |
| --- | --- | --- |
| *Number of patients* | 14 | 30 |
| *Sex distribution* | Male 71.4 %  Female 28.6% | Male 70.0 %  Female 30.0% |
| *Age (median/mean value)* | 59.5 [95% CI: 46.7-63.1] | 47.3 [95%CI: 41.9-52.6] |
| *Distribution according to the disease* | C81.1 – 7.1%  C82.7 – 42.9%  C83.3 – 42.9%  C84.5 – 7.1% | C81.0 – 3.3%  C81.1 – 30.0%  C81.2 – 3.3%  C82.2 – 3.3%  C82.7 – 3.3%  C83.3 – 43.3%  C84.4 – 10.0%  C90.0 – 3.3% |
| *Years lived with disease* | 1.0 [95%CI: 0.0-1.0] | 1.0 [95%CI: 1.0-1.0] |
| *Previous therapy* | ABVD, R-CHOP, R-DHAP, R-VIHA, EPOCH, R-ICE, R-ESHAP, Polivy-R-B | ABVD, OEPA, BEACOPP, escBEACOPP, DHAP, IGEV, BV-AVD, R-CHOP, R-EPOCH, R-CHOEP, R-Polivy-CHP, Pola-R-CHP+rituximab, DA-EPOCH+Bortezomib |
| *Average number of cycles with previous therapy* | 5.8 [95% CI: 4.0-11.5] | 6.0 [95%CI 4.0-7.8] |
| *Interval diagnosis–Pt-based therapy (years)* | 1.0 [95%CI: 0.0-1.0] | 1.0 [95%CI: 1.0-1.0] |

**Legend***: C81.0 Classical Hodgkin lymphoma; C81.1 Hodgkin disease, lymphocyte-rich type, stage IIA; C81.2 Hodgkin lymphoma, mixed cellularity; C82.2 Follicular lymphoma, large cell; C82.7 Follicular lymphoma, grade 3A, stage IV; C83.3 Diffuse large B-cell lymphoma; C84.4 Non-Hodgkin lymphoma — anaplastic large cell, T-cell type; C84.5 Other and unspecified T-cell lymphomas; C90.0 Plasmablastic large cell B-cell lymphoma;*

*ABVD: doxorubicin, bleomycin, vinblastine, and dacarbazine; R-CHOP: R- rituximab, C- cyclophosphamide, H – doxorubicin, O – vincristine, P- prednisolone; R-DHAP: R – rituximab (a targeted therapy) D – dexamethasone (a steroid) HA – high-dose cytarabine (Ara-C – a chemotherapy) P – cisplatin (platinum – a chemotherapy); R-VIHA: mabthera, vinorelbin, ifosfamide, mesna, cytarabine; EPOCH: etoposide, prednisone, vincristine, cyclophosphamide, doxorubicin; R-ICE: ifosfamde, carboplatin, etoposide+/-rituximab; R-ESHAP: rituximab+etoposide+methylprednisolone+cytarabine+cisplatin; Polivy-R-B – polatuzumab, rituximab, bendamustin; OEPA:vincristine+etoposide+prednisone+doxorubicin; BEACOPP: bleomycin, etoposide, doxorubicin, cyclophosphamide, vincristine, procarbazine, and prednisone; IGEV: gemcitabine, vinorelbin, ifosfamide, mesna, furosemide; BV-AVD: brentuximab vedotin DOXOrubicin vinBLASTine dacarbazine; R-EPOCH:* *etoposide phosphate, prednisone, vincristine sulfate (Oncovin), cyclophosphamide, doxorubicin hydrochloride (hydroxydaunorubicin), and rituximab; R-CHOEP:* *rituximab, cyclophosphamide, doxorubicin, vincristine, and etoposide; R-Polivy-CHP* *polatuzumab vedotin with standard R-CHOP; Pola-R-CHP: polatuzumab vedotin, cyclophosphamide, doxorubicin, prednisone, rituximab; DA-EPOCH+bortezomib: dose-adjusted etoposide, prednisone, vincristine, cyclophosphamide, doxorubicin, bortezomib.*

## **Clinical trials data**

**Table S9.** Clinical trials with Pt-based drugs for the period 2022-2025 identified in ClinicalTrials.gov

| **ClinicalTrials.gov identifier** | **Pt-based agent** | **Study design** | **Phase** | **Study population/condition** | **ICD-O category** | **Objective** | **Intervention(s)** | **Study period; country** |
| --- | --- | --- | --- | --- | --- | --- | --- | --- |
| NCT04397003 | carboplatin | Single-group assignment open-label study | Phase 2 | Extensive stage small-cell lung cancer (ES-SCLC) | C30–C39: respiratory system and intrathoracic organs | To evaluate the efficacy and/or safety of a combination regimen | Neoantigen DNA vaccine + carboplatin/etoposide/durvalumab + durvalumab | 2022–2029; USA |
| NCT04575961 | carboplatin | Single-arm, multicenter study | Phase 2 | Low-grade ovarian cancer | C51–C58: female genital organs | To evaluate the efficacy and/or safety of a combination regimen | Pembrolizumab + platinum-based chemotherapy (investigator's choice: carboplatin + gemcitabine or carboplatin + pegylated liposomal doxorubicin) | 2022–2028; Germany |
| NCT04929392 | carboplatin | Single-group assignment open-label study | Phase 2 | Esophageal or esophageal/gastroesophageal junction cancer | C15–C26: digestive organs | To evaluate the efficacy and/or safety of a combination regimen | Chemoradiation (including carboplatin), pembrolizumab, lenvatinib | 2022–2027; USA |
| NCT04943445 | carboplatin | Prospective, single-arm, multi-institutional, open-label study | Phase 2 | Locally advanced squamous cell carcinoma of the larynx | C30–C39: respiratory system and intrathoracic organs | To evaluate the efficacy and/or safety of a combination regimen | Chemo-immunotherapy (carboplatin + paclitaxel + pembrolizumab) + radiation therapy | 2022–2025; Brazil |
| NCT05173987 | carboplatin | Randomized, open-label, active-comparator controlled study | Phase 3 | Mismatch repair deficient (dMMR) advanced or recurrent endometrial carcinoma who have not previously been treated with prior systemic chemotherapy | C51–C58: female genital organs | To evaluate the efficacy and/or safety of a combination regimen | Pembrolizumab vs. carboplatin + paclitaxel | 2022–2027; USA |
| NCT05181462 | carboplatin | Randomized non-comparative open-label study | Phase 1b/2 | Advanced or metastatic triple-negative breast cancer | C50: breast | To evaluate the efficacy and/or safety of a combination regimen | Carboplatin + gemcitabine + nadunolimab vs. carboplatin + gemcitabine | 2022–2026; Spain |
| NCT05184712 | carboplatin | Randomized, double-blind, multicenter study | Phase 3 | EGFR-mutant locally advanced or metastatic non-squamous non-small-cell lung cancer | C30–C39: respiratory system and intrathoracic organs | To evaluate the efficacy and/or safety of a combination regimen | Ivonescimab + pemetrexed + carboplatin vs. placebo + pemetrexed + carboplatin | 2022–2025; China |
| NCT05200481 | carboplatin | Randomized parallel-group open-label non-comparative multicenter study | Phase 2 | Advanced ALK-positive non-small-cell lung cancer | C30–C39: respiratory system and intrathoracic organs | To evaluate the efficacy and/or safety of a combination regimen | Brigatinib alone vs. brigatinib + carboplatin + pemetrexed | 2022–2028; France |
| NCT05221645 | carboplatin | Open-label, multicenter, randomized study | Phase 2 | Relapsed or refractory diffuse large B-cell lymphoma | C42: hematopoietic and reticuloendothelial systems | To evaluate the efficacy and/or safety of a combination regimen | Rituximab + ifosfamide + carboplatin + etoposide (R-ICE) + autologous stem cell transplant vs. pembrolizumab + R-ICE + autologous stem cell transplant | 2022–2026; UK |
| NCT05244213 | carboplatin | Single-arm, open-label single-center study | Phase 2 | EGFR-mutant stage IIB-IIIB NSCLC | C30–C39: respiratory system and intrathoracic organs | To evaluate the efficacy and/or safety of a combination regimen | Sintilimab + doublet platinum-based chemotherapy (carboplatin and nab-paclitaxel) | 2022–2025; Japan |
| NCT05252416 | carboplatin | Open-label, first-in-human study | Phase 1/2 | Advanced solid tumors | C15–C26: digestive organs | To evaluate safety, tolerability, pharmacokinetics, pharmacodynamics of a new drug in combination and as monotherapy | BLU-222 monotherapy, BLU-222 + carboplatin, BLU-222 + ribociclib + fulvestrant | 2022–2026; USA |
| NCT05258279 | carboplatin | Not reported | Phase 2 | Advanced nonsquamous non-small-cell lung cancer harboring EGFR mutations | C30–C39: respiratory system and intrathoracic organs | To evaluate the efficacy and/or safety of a combination regimen | Pemetrexed + carboplatin + pembrolizumab + lenvatinib | 2022–2024; Japan |
| NCT05276973 | carboplatin | Not reported | Phase 1/1b | Stage III or IV epithelial ovarian cancer | C51–C58: female genital organs | To evaluate the safety and dose of a combination regimen | Paclitaxel, carboplatin, ipatasertib | 2022–2026; USA |
| NCT05329025 | carboplatin | Non-randomized single-group assignment open-label study | Phase 2 | Stage IIIB/C and stage IV Non-Small Cell Lung Cancer (NSCLC) | C30–C39: respiratory system and intrathoracic organs | To evaluate the efficacy and/or safety of a combination regimen | QL1706 + bevacizumab, paclitaxel or pemetrexed, carboplatin | 2022–2024; China |
| NCT05361395 | carboplatin | Parallel-group open-label non-randomized study | Phase 1b | Extensive stage small-cell lung | C30–C39: respiratory system and intrathoracic organs | To evaluate the efficacy and/or safety of a combination regimen | Tarlatamab + carboplatin, etoposide, + PD-L1 Inhibitor | 2022–2028; USA |
| NCT05364424 | carboplatin | Open-label, multicenter, single-arm study | Phase 1b | Relapsed/refractory transplant or CAR-T therapy eligible diffuse B-cell lymphoma | C42: hematopoietic and reticuloendothelial systems | To evaluate the efficacy and/or safety of a combination regimen | Glofitamab + rituximab, ifosfamide, carboplatin, and etoposide | 2022–2026; USA |
| NCT05381909 | carboplatin | Open-label randomized parallel-group study | Phase 1 | Advanced or spreading solid tumors | Not specified | To evaluate the efficacy and/or safety of a combination regimen | BGB-24714 as monotherapy, in combination with carboplatin + paclitaxel | 2022–2026; USA |
| NCT05456685 | carboplatin | Multicenter, open-label study sequential-assignment | Phase 2 | Folate receptor alpha (FRα) expressing, platinum-sensitive epithelial ovarian cancer | C51–C58: female genital organs | To evaluate the efficacy and/or safety of a combination regimen | MIRV + carboplatin | 2022–2026; USA |
| NCT05488873 | carboplatin | Randomized, double-blind, placebo-controlled adaptive study | Phase 2 | Chemotherapy-induced (carbo+paclitaxel) peripheral neuropathy | Not specified | To evaluate a strategy for reducing treatment-related toxicity | Placebo vs. WST-057 active | 2022–2026; USA |
| NCT05605535 | carboplatin | Double-blind, placebo-controlled, multicenter study | Phase 2 | Newly diagnosed advanced ovarian cancer | C51–C58: female genital organs | To evaluate the efficacy and/or safety of a combination regimen | Oregovomab + chemotherapy (paclitaxel + carboplatin) vs. placebo + chemotherapy | 2023–2027; India |
| NCT05659251 | carboplatin | Prospective, single-arm, single-center, exploratory study | Phase 2 | Resectable esophageal squamous cell carcinoma | C15–C26: digestive organs | To evaluate the efficacy and/or safety of a combination regimen | Serplulimab + albumin-bound paclitaxel, carboplatin, neoadjuvant therapy | 2022–2029; China |
| NCT05746481 | carboplatin | Single-group assignment open-label study | Phase 2 | Metastatic non-squamous NSCLC patients with asymptomatic untreated brain metastases | C30–C39: respiratory system and intrathoracic organs | To evaluate the efficacy and/or safety of a combination regimen | Tiragolumab + atezolizumab, pemetrexed, and carboplatin | 2023–2026; USA |
| NCT05756972 | carboplatin | Single-arm study | Phase 2 | EGFR-mutant locally advanced or metastatic non-squamous NSCLC | C30–C39: respiratory system and intrathoracic organs | To evaluate the efficacy and/or safety of a combination regimen | PM8002 + chemotherapy (pemetrexed and carboplatin) | 2023–2025; China |
| NCT05775289 | carboplatin | Randomized, multicenter, double-blind, controlled study | Phase 2 | Previously untreated, locally advanced, unresectable (Stage IIIB/IIIC) or metastatic (Stage IV) non-small-cell lung cancer (NSCLC) | C30–C39: respiratory system and intrathoracic organs | To evaluate safety, efficacy and pharmacokinetic characteristics of a combination | Tobemstomig (RO7247669) + platinum-based chemotherapy (paclitaxel + pemetrexed + carboplatin) vs. pembrolizumab + platinum-based chemotherapy (paclitaxel + pemetrexed + carboplatin) | 2023–2026; USA |
| NCT05840016 | carboplatin | Randomized, controlled, multicenter study | Phase 3 | IIIB/C (unsuitable for radical therapy) or stage IV squamous non-small-cell lung cancer | C30–C39: respiratory system and intrathoracic organs | To evaluate the efficacy and/or safety of a combination regimen | AK112 + carboplatin, paclitaxel vs. PD-1 inhibitor + carboplatin, paclitaxel | 2023–2026; China |
| NCT05889390 | carboplatin | Pivotal, randomized (1:1), open-label, two-treatment group, single-center trial | Phase 2/3 | HER2-negative, stage II-III breast cancer | C50: breast | To evaluate the efficacy and/or safety of a combination regimen | WTAX ( + carboplatin) + AC neoadjuvant chemotherapy protocol + mEHT vs. wTAX ( + carboplatin) + AC neoadjuvant chemotherapy protocol | 2023–2025; Hungary |
| NCT06057038 | carboplatin | Not reported | Phase 1 | Non-central nervous system (non-CNS) malignant solid tumours | Not specified | To evaluate safety, efficacy and pharmacokinetic characteristics of a combination | Biological GEN1042 + pembrolizumab or pembrolizumab + standard of care (SOC) chemotherapy (cisplatin/carboplatin + 5-FU) | 2023–2026; Japan |
| NCT06107868 | carboplatin | Not reported | Phase 1 | TP53 mutated ovarian or uterine cancer | C51–C58: female genital organs | To evaluate the safety and dose of a combination regimen | RP6306 + carboplatin, paclitaxel, | 2024–2026; Canada |
| NCT06110195 | carboplatin | Not reported | Phase 1 | Locoregionally advanced squamous cell carcinoma of the head and neck | C00–C14: lip, oral cavity, and pharynx | To evaluate the safety and dose of a combination regimen | Xevinapant + carboplatin, paclitaxel, and radiation (chemoRT) | 2024–2027; USA |
| NCT06236425 | carboplatin | Open-label study | Phase 1b | Advanced HNSCC who have initially progressed on pembrolizumab or pembrolizumab/platinum chemotherapy | C00–C14: lip, oral cavity, and pharynx | To evaluate safety and tolerability of a combination | TIL harvest/standard of care treatment (cisplatin/carboplatin or pembrolizumab) vs. experimental (TBio-4101) | 2024–2026; USA |
| NCT06321068 | carboplatin | Not reported | Phase 2/3 | Advanced or recurrent mismatch repair protein-deficient (dMMR) endometrial cancer | C51–C58: female genital organs | To evaluate the efficacy and/or safety of a combination regimen | BAT1308 + platinum-containing chemotherapy (carboplatin) vs. placebo + platinum-containing chemotherapy | 2024–2025; China |
| NCT06396065 | carboplatin | Randomized, double-blind, multicenter study | Phase 3 | Locally advanced or metastatic non-squamous non-small-cell lung cancer | C30–C39: respiratory system and intrathoracic organs | To evaluate the efficacy and/or safety of a combination regimen | Ivonescimab + pemetrexed + carboplatin vs. placebo + pemetrexed + carboplatin | 2023–2025; USA |
| NCT06449209 | carboplatin | Multicenter, open-label, parallel group study | Phase 2 | Small-cell lung cancer | C30–C39: respiratory system and intrathoracic organs | To evaluate the efficacy and/or safety of a combination regimen | Cohort 1, Cohort 2/Cohort 3 takes different drugs: BNT327 DL1 + carboplatin + etoposide; BNT327 DL1 + paclitaxel | 2024–2028; USA |
| NCT06888531 | carboplatin | Prospective, multicenter, randomized controlled trial | Phase 2 | Locally advanced resectable esophageal squamous cell carcinoma | C15–C26: digestive organs | To evaluate the efficacy and/or safety of a combination regimen | Neoadjuvant chemoradiotherapy (including carboplatin) combined with PD-1/PD-L1 inhibitors | 2025–2027; China |
| NCT05704985 | carboplatin/oxaliplatin | Sequential-assignment non-randomized open-label study | Phase 1 | Inoperable Locally Advanced and/or Metastatic EGFR+ Tumors With Progressive Disease Failing Systemic Therapy | C30–C39: respiratory system and intrathoracic organs | To evaluate the efficacy and/or safety of a combination regimen | DK210 (EGFR) as monotherapy, in combination with chemotherapy (including carboplatin/oxaliplatin) | 2023–2025; USA |
| NCT04402073 | cisplatin | Comparative, randomized open-label study | Phase 2 | SHH-subgroup (sonic hedgehog- subgroup) of medulloblastoma (a rare brain cancer) | C69–C72: eye, brain, and other parts of the central nervous system | To evaluate the efficacy and/or safety of a combination regimen | Radiotherapy + cisplatin, lomustine, vincristine vs. sonidegib + cisplatin + lomustine + vincristine + radiotherapy | 2022–2025; Australia |
| NCT04630730 | cisplatin | Single-group assignment prospective single-arm open-label multicenter study | Phase 2 | Localized muscle-invasive bladder cancer | C64–C68: urinary tract | To evaluate the efficacy and/or safety of a combination regimen | Recombinant intravesical BCG (Bacillus Calmette-Guérin VPM1002BC) immuno-stimulating agent + atezolizumab + standard neoadjuvant chemotherapy cisplatin/gemcitabine | 2022–2030; Switzerland |
| NCT04719988 | cisplatin | Single-group assignment open-label study | Phase 2 | Squamous cell carcinoma of the anus stage III (rare cancer) | C15–C26: digestive organs | To evaluate the efficacy and/or safety of a combination regimen | Modified DCF (docetaxel, cisplatin, 5-FU) + Ezabenlimab followed by radiotherapy | 2022–2026; France |
| NCT05186974 | cisplatin | Open-label, multicenter study | Phase 2 | Advanced or metastatic non-small-cell lung cancer (NSCLC) | C30–C39: respiratory system and intrathoracic organs | To evaluate the efficacy and/or safety of a combination regimen | Sacituzumab Govitecan Combinations (cisplatin; pembrolizumab + cisplatin) | 2022–2026; USA |
| NCT05193617 | cisplatin | Randomized parallel-group open-label study | Phase 2 | Locoregionally advanced nasopharyngeal carcinoma | C30–C39: respiratory system and intrathoracic organs | To evaluate the efficacy and/or safety of a combination regimen | GP + penpulimab (PD-1 monoclonal antibody) and anlotinib (TKI) neoadjuvant therapy + CCRT + penpulimab adjuvant therapy vs. GP + penpulimab neoadjuvant therapy + CCRT + penpulimab adjuvant therapy | 2022–2027; China |
| NCT05211232 | cisplatin | Double-blind, placebo-controlled study | Phase 3 | Locoregionally advanced nasopharyngeal carcinoma | C30–C39: respiratory system and intrathoracic organs | To evaluate the efficacy and/or safety of a combination regimen | Gemcitabine plus cisplatin (GP) + tislelizumab neoadjuvant therapy + CCRT + tislelizumab adjuvant therapy vs. GP combine with Placebo neoadjuvant therapy + CCRT + Placebo adjuvant therapy | 2022–2028; China |
| NCT05211323 | cisplatin | Randomized parallel-group open-label study | Phase 2 | Unresectable or advanced liver cancer | C15–C26: digestive organs | To evaluate the efficacy and/or safety of a combination regimen | Atezolizumab, bevacizumab, gemcitabine, cisplatin vs. atezolizumab, gemcitabine, cisplatin | 2022–2025; USA |
| NCT05235516 | cisplatin | Randomized, double-blind, placebo-controlled study | Phase 3 | Locally advanced cervical cancer | C51–C58: female genital organs | To evaluate the efficacy and/or safety of a combination regimen | AK104 + radiation: EBRT + radiation: BT + Drug: cisplatin vs. placebo + radiation: EBRT + radiation: BT + drug: cisplatin | 2022–2029; China |
| NCT05294172 | cisplatin | Randomized, double-blind, placebo-controlled and multicenter study | Phase 3 | Recurrent or metastatic nasopharyngeal carcinoma | C30–C39: respiratory system and intrathoracic organs | To evaluate the efficacy and/or safety of a combination regimen | KL-A167 + gemcitabine + cisplatin vs. placebo + gemcitabine + cisplatin | 2022–2027; China |
| NCT05340270 | cisplatin | Multicenter, randomized controlled study | Phase 2 | Locoregionally advanced nasopharyngeal carcinoma | C30–C39: respiratory system and intrathoracic organs | To evaluate the efficacy and/or safety of a combination regimen | PD-1 inhibitor + GP chemotherapy (gemcitabine + cisplatin) vs. GP chemotherapy (gemcitabine + cisplatin) | 2022–2026; China |
| NCT05513573 | cisplatin | Randomized, double-blind, multicenter study | Phase 2 | Recurrent or metastatic nasopharyngeal carcinoma (NPC) | C30–C39: respiratory system and intrathoracic organs | To evaluate the efficacy and/or safety of a combination regimen | HLX07 (Recombinant Anti-EGFR Humanized Monoclonal Antibody Injection) + serplulimab (HLX10, Recombinant Anti-PD-1 Humanized Monoclonal Antibody) + chemotherapy vs. placebo + serplulimab + chemotherapy | 2022–2026; China |
| NCT05520619 | cisplatin | Randomized parallel-group open-label study | Phase 2 | Locally advanced esophageal squamous cell carcinoma (ESCC) | C15–C26: digestive organs | To evaluate the efficacy and/or safety of a combination regimen | Tislelizumab plus chemoradiotherapy with maintenance vs. tislelizumab plus CRT without maintenance | 2022–2026; China |
| NCT05576272 | cisplatin | Randomized, open-label, multicenter study | Phase 2/3 | Recurrent or metastatic nasopharyngeal carcinoma | C30–C39: respiratory system and intrathoracic organs | To evaluate the efficacy and/or safety of a combination regimen | QL1706 + gemcitabine + cisplatin vs. carrilizumab + gemcitabine + cisplatin | 2022–2024; China |
| NCT05587374 | cisplatin | Randomized parallel-group open-label study | Phase 3 | High-risk locoregionally-advanced nasopharyngeal carcinoma (LANPC) | C30–C39: respiratory system and intrathoracic organs | To evaluate the efficacy and/or safety of a combination regimen | Cadonilimab + gemcitabine + cisplatin + intensity-modulated radiotherapy vs. gemcitabine + cisplatin + intensity-modulated radiotherapy | 2023–2029; China |
| NCT05628233 | cisplatin | Multicenter, randomized, controlled, two-arm, open-label study | Phase 2 | Hearing loss ototoxic | Not specified | To evaluate a strategy for reducing treatment-related toxicity | Cisplatin-based chemotherapy without receiving r-azasetron besylate vs. cisplatin-based chemotherapy receiving r-azasetron besylate | 2022–2025; France |
| NCT05712356 | cisplatin | Double-blind, placebo-controlled, multicenter, randomized study | Phase 2a | Advanced solid tumours | Not specified | To evaluate the efficacy and/or safety of a combination regimen | LSTA1 + SoC vs. SoC alone (oxaliplatin/cisplatin) | 2023–2030; USA |
| NCT05776758 | cisplatin | Randomized parallel-group open-label study | Phase 3 | Muscle-invasive bladder carcinoma | C64–C68: urinary tract | To demonstrate the non-inferiority of both regimen | Radical cystectomy (RC) alone vs. cisplatin-based neoadjuvant chemotherapy plus radical cystectomy | 2024–2027; Italy |
| NCT05781958 | cisplatin | Single-group assignment open-label study | Phase 2 | Advanced intrahepatic cholangiocarcinoma | C15–C26: digestive organs | To evaluate the efficacy and/or safety of a combination regimen | Cadonilimab + Gem/cisplatin | 2022–2024; China |
| NCT05941741 | cisplatin | Randomized parallel-group open-label study | Phase 3 | Nasopharyngeal carcinoma | C30–C39: respiratory system and intrathoracic organs | To evaluate the efficacy and/or safety of a combination regimen | Induction chemotherapy (IC) gemcitabine and cisplatin + low-dose radiation and immune checkpoint inhibitor (ICI) cadonilimab followed by concurrent chemoradiotherapy (CCRT) versus IC + CCRT | 2024–2029; China |
| NCT06009861 | cisplatin | Single-group assignment | Phase 2 | Oral squamous cell carcinoma, oropharyngeal squamous cell carcinoma | C00–C14: lip, oral cavity, and pharynx | To evaluate the efficacy and/or safety of a combination regimen | Neoadjuvant chemoimmunotherapy: tislelizumab, albumin-bound paclitaxel + cisplatin | 2023–2027; China |
| NCT06010303 | cisplatin | Randomized, open-label study | Phase 2 | Unresectable locally advanced or metastatic esophageal squamous cell carcinoma | C15–C26: digestive organs | To evaluate the efficacy and/or safety of a combination regimen | LBL-007 in combination with tislelizumab plus chemotherapy doublet vs. tislelizumab and chemotherapy (cisplatin + 5-fluorouracil or cisplatin + paclitaxel) | 2023–2026; China |
| NCT06019520 | cisplatin | Randomized parallel-group open-label study | Not applicable | Non-haematological malignancies (breast, ovary and head and neck squamous cell carcinomas) | Not specified | To evaluate a strategy for reducing treatment-related toxicity | N-Acetylcysteine + cisplatin-based chemotherapy vs. cisplatin-based chemotherapy | 2023–2024; Pakistan |
| NCT06145412 | cisplatin | Single-group assignment | Phase 2 | High-risk head and neck cancers | C00–C14: lip, oral cavity, and pharynx | To evaluate the efficacy and/or safety of a combination regimen | Xevinapant + post-operative cisplatin + radiotherapy | 2023–2026; USA |
| NCT06664983 | cisplatin/lobaplatin | Randomized study | Phase 3 | anti-PD-1 resistant recurrent or metastatic nasopharyngeal carcinoma | C30–C39: respiratory system and intrathoracic organs | To evaluate the efficacy and/or safety of a combination regimen | TPC chemotherapy vs. Cadonilimab with TPC chemotherapy | 2024–2026; China |
| NCT06762158 | cisplatin | Single-group assignment | Not applicable | Locally advanced oesophageal squamous cell cancer | C15–C26: digestive organs | To evaluate the efficacy and/or safety of a combination regimen | Neoadjuvant chemoradiotherapy + perioperative PD-1 inhibitor toripalimab | 2024–2029; China |
| NCT06771596 | cisplatin | Single-arm study single-group assignment open-label study | Phase 2 | High-risk, locally advanced squamous cell carcinoma of the cervix | C51–C58: female genital organs | To evaluate the efficacy and/or safety of a combination regimen | Nimotuzumab + chemoradiotherapy | 2025; China |
| NCT05063552 | cisplatin/carboplatin | Not reported | Phase 2/3 | Recurrent or metastatic or advanced stage head and neck cancer | C00–C14: lip, oral cavity, and pharynx | To evaluate the efficacy and/or safety of a combination regimen | Standard therapy (chemotherapy plus cetuximab) vs. bevacizumab + standard chemotherapy vs. bevacizumab + atezolizumab | 2023–2027; USA |
| NCT05092412 | cisplatin/carboplatin | A first-line multicenter, single-arm exploratory study | Phase 2 | Extensive-stage small-cell lung cancer | C30–C39: respiratory system and intrathoracic organs | To evaluate the efficacy and/or safety of a combination regimen | Durvalumab, etoposide, and cisplatin/carboplatin | 2022–2027; China |
| NCT05116462 | cisplatin/carboplatin | Randomized, double-blind study | Phase 3 | Resectable stage II to IIIB (resectable N2 only) non-small-cell lung cancer (NSCLC) | C30–C39: respiratory system and intrathoracic organs | To evaluate the efficacy and/or safety of a combination regimen | Sintilimab plus platinum-based chemotherapy vs. placebo plus platinum-based chemotherapy | 2024–2028; China |
| NCT05180799 | cisplatin/carboplatin | Non-randomized parallel-group open-label study | Phase 1/2 | Melanoma - 1L; nonsquamous or recurrent NSCLC (type IIB, IIIA, IV) with single or any combination of the following mutations: KRAS mutation STK11 mutation KEAP1 mutation PD-L1 tumor proportion score (TPS) <1% | C30–C39: respiratory system and intrathoracic organs | To evaluate safety, efficacy and pharmacokinetic characteristics of a combination | BA3071 in different combinations ( + pemetrexed + cisplatin/carboplatin; + nivolumab + pembrolizumab) | 2022–2026; USA |
| NCT05224141 | cisplatin/carboplatin | Randomized, double-blind study | Phase 3 | Extensive-stage small-cell lung cancer | C30–C39: respiratory system and intrathoracic organs | To evaluate the superiority of the investigational combination | Pembrolizumab + vibostolimab + etoposide + platinum vs. atezolizumab + etoposide + platinum | 2022–2027; USA |
| NCT05226598 | cisplatin/carboplatin | Randomized, double-blind study | Phase 3 | Treatment-naïve metastatic participants with non-small-cell lung cancer (NSCLC) | C30–C39: respiratory system and intrathoracic organs | To evaluate the superiority of the investigational combination | Pembrolizumab/vibostolimab + carboplatin/cisplatin + paclitaxel + nab-paclitaxel + pemetrexed vs. pembrolizumab + carboplatin/cisplatin + paclitaxel + nab-paclitaxel + pemetrexed | 2022–2026; USA |
| NCT05298423 | cisplatin/carboplatin | Open-label study | Phase 3 | Unresectable, locally advanced, stage III NSCLC | C30–C39: respiratory system and intrathoracic organs | To evaluate the efficacy and/or safety of a combination regimen | Pembrolizumab/vibostolimab coformulation + chemotherapy (platinum doublet) + radiotherapy vs. chemotherapy (platinum doublet) + radiotherapy + durvalumab | 2022–2026; USA |
| NCT05338970 | cisplatin/carboplatin | Randomized, open-label study | Phase 3 | Epidermal growth factor receptor mutated (EGFRm) non-small-cell lung cancer (NSCLC) | C30–C39: respiratory system and intrathoracic organs | To evaluate the efficacy and/or safety of a combination regimen | Patritumab deruxtecan vs. platinum-based chemotherapy (pemetrexed + cisplatin /carboplatin) | 2022–2026; USA |
| NCT05353257 | cisplatin/carboplatin | Randomized, double-blind, multicenter study | Phase 3 | Limited-stage small-cell lung cancer | C30–C39: respiratory system and intrathoracic organs | To evaluate the efficacy and/or safety of a combination regimen | Serplulimab + carboplatin/cisplatin-etoposide + radiotherapy vs. placebo + carboplatin/cisplatin-etoposide + radiotherapy | 2022–2026; USA |
| NCT05386888 | cisplatin/carboplatin | Single-group assignment | Phase 2 | Unresectable, locally advanced, stage III non-small-cell lung cancer (NSCLC) | C30–C39: respiratory system and intrathoracic organs | To evaluate the efficacy and/or safety of a combination regimen | GFH018 + toripalimab + standard chemo-radiotherapy (paclitaxel; carboplatin; cisplatin; pemetrexed; radiation: thoracic radiation therapy (TRT)) | 2022–2025; China |
| NCT05407675 | cisplatin/carboplatin | Non-randomized sequential-assignment open-label study | Phase 1/2 | Advanced solid tumors | Not specified | To evaluate safety of new combination | BMS-986408 in combination with nivolumab/nivolumab + ipilimumab/ nivolumab + Platinum-doublet chemotherapy | 2022–2025; USA |
| NCT05470595 | cisplatin/carboplatin | Single-arm study | Phase 2 | Advanced large-cell neuroendocrine cancer of the lung | C30–C39: respiratory system and intrathoracic organs | To evaluate the efficacy and/or safety of a combination regimen | Atezolizumab/cisplatin or carboplatin /etoposide | 2022–2029; Germany |
| NCT05566041 | cisplatin/carboplatin | Controlled, open-label, global randomized study crossover assignment | Phase 3 | Small cell lung cancer | C30–C39: respiratory system and intrathoracic organs | To evaluate the efficacy and/or safety of a combination regimen | RRx-001 + cisplatin/carboplatin + etoposide vs. cisplatin/carboplatin + etoposide | 2022–2025; USA |
| NCT05635708 | cisplatin/carboplatin | Open-label, multi-arm study | Phase 2 | Non-small-cell lung cancer | C30–C39: respiratory system and intrathoracic organs | To evaluate the efficacy and/or safety of a combination regimen | Tislelizumab + investigational agent(s) with or without chemotherapy (cisplatin/carboplatin/paclitaxel) | 2023–2025; USA |
| NCT05664971 | cisplatin/carboplatin | Non-randomized single-group assignment open-label study | Phase 1b/2 | Advanced lung cancer | C30–C39: respiratory system and intrathoracic organs | To evaluate safety, efficacy and pharmacokinetic characteristics of a combination | JS004 injection + toripalimab with or without standard chemotherapy | 2023–2025; China |
| NCT05687266 | cisplatin/carboplatin | Randomized, open-label, multicenter study | Phase 3 | Stage IIIB, IIIC, or IV NSCLC without actionable genomic alterations | C30–C39: respiratory system and intrathoracic organs | To evaluate the efficacy and/or safety of a combination regimen | Datopotamab deruxtecan + durvalumab + carboplatin vs. histologic-specific therapy (carboplatin, pembrolizumab, cisplatin, pemetrexed, paclitaxel) | 2022–2027; USA |
| NCT05722015 | cisplatin/carboplatin | Randomized, open-label study | Phase 3 | Metastatic non-small-cell lung cancer | C30–C39: respiratory system and intrathoracic organs | To evaluate safety, efficacy and pharmacokinetic characteristics of a combination | S.c. pembrolizumab with hyaluronidase (MK-3475A) vs. i.v. pembrolizumab + chemotherapy | 2023–2028; USA |
| NCT05765032 | cisplatin/carboplatin | Single-group assignment open-label study | Phase 1/2 | Advanced solid tumours | Not specified | To evaluate the efficacy and/or safety of a combination regimen | SHR-A1921 in combination with other anti-cancer agents (cisplatin, carboplatin) | 2022–2026; China |
| NCT05822934 | cisplatin/carboplatin | Randomized parallel-group study | Phase 3 | Muscle invasive urinary bladder cancer | C64–C68: urinary tract | To compare the efficacy of two platinum-based regimens | Carboplatin-gemcitabine vs. cisplatin-gemcitabine | 2022–2024; Egypt |
| NCT05975307 | cisplatin/carboplatin | Single-group assignment | Phase 2 | Muscle-invasive bladder carcinoma | C64–C68: urinary tract | To evaluate the efficacy and/or safety of a combination regimen | Chemotherapy (gemcitabine, cisplatin/carboplatin) plus anti-PD-1 therapy (toripalimab), followed by radiotherapy plus concurrent anti-PD-1 therapy | 2023–2029; China |
| NCT06091943 | cisplatin/carboplatin | Interventional open-label sequential-assignment | Phase 1 | Advanced or metastatic non-small-cell lung cancer | C30–C39: respiratory system and intrathoracic organs | To evaluate safety, efficacy and pharmacokinetic characteristics of a combination | Tislelizumab plus histology-based chemotherapy | 2023–2026; China |
| NCT06212752 | cisplatin/carboplatin | Randomized study | Phase 3 | Metastatic non-small-cell lung cancer | C30–C39: respiratory system and intrathoracic organs | To evaluate safety, efficacy and pharmacokinetic characteristics of a combination | Pembrolizumab with berahyaluronidase alfa plus platinum-doublet chemotherapy vs. intravenous pembrolizumab plus platinum-doublet chemotherapy | 2023–2028; Japan |
| NCT06237257 | cisplatin/carboplatin | Single-group assignment | Phase 2 | Locally advanced cervical cancer | C51–C58: female genital organs | To evaluate safety, efficacy and pharmacokinetic characteristics of a combination | SHR-1316 + concurrent chemoradiotherapy | 2024–2025; China |
| NCT06419673 | cisplatin/carboplatin | Prospective, multicenter, randomized, open-label controlled clinical trial | Phase 2 | Stage III or IVA cervical squamous cell carcinoma, adenocarcinoma, and adenosquamous cell carcinoma | C51–C58: female genital organs | To evaluate the efficacy and/or safety of a combination regimen | Serplulimab + chemoradiotherapy vs. concurrent chemoradiotherapy | 2024–2028; China |
| NCT06866951 | cisplatin/carboplatin | Randomized parallel-group open-label study | Phase 2/3 | Cervical cancer patients with positive lymph nodes after surgery | C51–C58: female genital organs | To evaluate the efficacy and/or safety of a combination regimen | Chemotherapy + immunotherapy vs. chemoradiotherapy | 2025–2030; China |
| NCT07018622 | cisplatin/carboplatin | Randomized, double-blind, placebo controlled study | Phase 2 | With solid tumours | Not specified | To evaluate a strategy for reducing treatment-related toxicity | SGLT2-inh + cisplatin/carboplatin | 2025–2026; Mexico |
| NCT05771480 | cisplatin/carboplatin/oxaliplatin | Single-group assignment open-label study | Phase 3 | Advanced biliary tract cancers | C15–C26: digestive organs | To evaluate the efficacy and/or safety of a combination regimen | Durvalumab + gemcitabine based chemotherapy (with or without cisplatin/oxaliplatin/carboplatin) | 2023–2026; USA |
| NCT05945823 | cisplatin/oxaliplatin | Non-randomized parallel-group open-label study | Phase 2 | Solid tumors | Not specified | To evaluate the efficacy and/or safety of a combination regimen | Futibatinib in combination with PD-1 antibody-based SoC therapy | 2023–2027; USA |
| NCT04481204 | oxaliplatin | Parallel-group randomized open-label study | Phase 2 | Resectable, borderline resectable, or locally advanced pancreatic cancer | C15–C26: digestive organs | To evaluate the efficacy and/or safety of a combination regimen | (different groups and each has a control arm) – mFOLFIRINOX, radiation therapy | 2023–2027; USA |
| NCT05052801 | oxaliplatin | Parallel-group randomized double blind study | Phase 3 | Gastric cancer; gastroesophageal junction adenocarcinoma | C15–C26: digestive organs | To evaluate the efficacy and/or safety of a combination regimen | Bemarituzumab + mFOLFOX6 vs. Placebo + mFOLFOX6 | 2022–2025; USA |
| NCT05111626 | oxaliplatin | Not reported | Phase 1b/3 | Previously untreated advanced gastric and gastroesophageal junction cancer with FGFR2b overexpression | C15–C26: digestive organs | To evaluate the efficacy and/or safety of a combination regimen | Bemarituzumab + mFOLFOX6 and nivolumab; bemarituzumab + mFOLFOX6 or CAPOX and nivolumab; placebo + mFOLFOX6 or CAPOX and Nivolumab | 2022–2027; USA |
| NCT05180734 | oxaliplatin | Sequential-assignment quadruple study | Phase 3 | Gastric or esophagogastric junction adenocarcinoma | C15–C26: digestive organs | To evaluate the efficacy and/or safety of a combination regimen | Toripalimab + XELOX or SOX vs. Placebo + XELOX or SOX | 2022–2028; China |
| NCT05198609 | oxaliplatin | Parallel-group randomized open-label study | Phase 3 | Hepatocellular carcinoma with portal vein invasion | C15–C26: digestive organs | To evaluate the efficacy and/or safety of a combination regimen | Camrelizumab, apatinib plus FOLFOX-HAIC vs. camrelizumab and apatinib | 2022–2026; China |
| NCT05251948 | oxaliplatin | Randomized parallel-group open-label study | Phase 1/2 | Gastric and gastroesophageal junction carcinoma | C15–C26: digestive organs | To evaluate the efficacy and/or safety of a combination regimen | Atezolizumab + CAPOX (capecitabine + oxaliplatin) vs. atezo + CAPOX + tiragolumab | 2022–2026; China |
| NCT05329766 | oxaliplatin | Parallel-group randomized study | Phase 2 | Locally advanced unresectable or metastatic gastric, GEJ, and esophageal adenocarcinoma | C15–C26: digestive organs | To evaluate the efficacy and/or safety of a combination regimen | Domvanalimab and zimberelimab + FOLFOX; zimberelimab + FOLFOX | 2022–2027; USA |
| NCT05394740 | oxaliplatin | Open-label, single-arm, single-center study | Phase 1b/2 | Unresectable advanced/recurrent gastric/ gastroesophageal junction/esophageal adenocarcinoma | C15–C26: digestive organs | To evaluate the efficacy and/or safety of a combination regimen | Regorafenib, nivolumab + CapeOX/FOLFOX | 2022–2025; Japan |
| NCT05435313 | oxaliplatin | Single-center, single-arm, open-label study | Phase 2 | Colorectal liver metastases cancer (CRLM) who failed standard therapy | C15–C26: digestive organs | To evaluate the efficacy and/or safety of a combination regimen | Fruquintinib + tislelizumab and HAIC (TOMOX/TOMIRI – including oxaliplatin) | 2022–2024; China |
| NCT05480306 | oxaliplatin | Randomized, open-label, two-part, multicenter study | Phase 2 | Advanced colorectal cancer patients | C15–C26: digestive organs | To evaluate the efficacy and/or safety of a combination regimen | DKN-01 + FOLFIRI/FOLFOX + bevacizumab vs. FOLFIRI/FOLFOX + bevacizumab | 2022–2025; USA |
| NCT05504720 | oxaliplatin | Open-label, single-arm, multicenter study | Phase 2 | HER2 positive localized esophagogastric adenocarcinoma | C15–C26: digestive organs | To evaluate the efficacy and/or safety of a combination regimen | Pembrolizumab, trastuzumab + FLOT (including oxaliplatin) | 2023–2027; Germany |
| NCT05546853 | oxaliplatin | Multicenter prospective single-arm study | Phase 1b | Locally advanced pancreatic ductal adenocarcinoma | C15–C26: digestive organs | To evaluate the efficacy and/or safety of a combination regimen | NP137 + mFOLFIRINOX | 2023–2025; France |
| NCT05568095 | oxaliplatin | Randomized, open-label, multicenter study | Phase 3 | Untreated locally advanced unresectable or metastatic gastric, gastroesophageal junction, and esophageal adenocarcinoma | C15–C26: digestive organs | To evaluate the efficacy and/or safety of a combination regimen | Domvanalimab + zimberelimab + FOLFOX/CAPOX vs. nivolumab + FOLFOX/CAPOX | 2022–2027; USA |
| NCT05627635 | oxaliplatin | Not reported | Phase 1/2 | Microsatellite stable (MSS) colorectal cancer | C15–C26: digestive organs | To evaluate the safety, efficacy, and doses of a combination regimen | FOLFOX, bevacizumab, balstilimab, and botensilimab | 2023–2025; USA |
| NCT05671822 | oxaliplatin | Open-label, multicenter study | Phase 1b/2 | HER2 expression advanced/metastatic gastric/gastroesophageal junction adenocarcinoma patients | C15–C26: digestive organs | To evaluate the efficacy and/or safety of a combination regimen | SHR-A1811 + chemotherapy (including oxaliplatin) and/or immunotherapy | 2023–2026; China |
| NCT05864105 | oxaliplatin | Single-group assignment open-label study | Phase 2 | Inoperable hepatocellular carcinoma | C15–C26: digestive organs | To evaluate the efficacy and/or safety of a combination regimen | PM8002 + FOLFOX-4 | 2022–2026; China |
| NCT05867121 | oxaliplatin | Open-label, multicenter dose-expansion study | Phase 1b | Locally advanced or metastatic solid tumors such as non-small-cell lung cancer (NSCLC), gastric cancer (GC) and pancreatic ductal adenocarcinoma (PDAC) | Not specified | To evaluate the efficacy and/or safety of a combination regimen | Checkpoint inhibitor (CPI): RO7496353 + atezolizumab; RO7496353, nivolumab, and oxaliplatin; RO7496353 + atezolizumab + nab-paclitaxel + gemcitabine | 2023–2026; USA |
| NCT05941481 | oxaliplatin | Single-group assignment open-label study | Phase 2 | Gastric or gastroesophageal junction adenocarcinoma | C15–C26: digestive organs | To evaluate the efficacy and/or safety of a combination regimen | Immunotherapy tislelizumab + chemotherapy: tislelizumab + XELOX regimen (capecitabine + oxaliplatin) and concurrent radiotherapy | 2023–2026; China |
| NCT06094140 | oxaliplatin | Single-group assignment open-label study | Phase 2 | Resectable or borderline resectable pancreatic adenocarcinoma | C15–C26: digestive organs | To evaluate the safety, efficacy, and doses of a combination regimen | Durvalumab + mFOLFIRINOX | 2022–2026; Australia |
| NCT06106308 | oxaliplatin | Randomized, open-label study | Phase 2 | Metastatic colorectal cancer in patients with a KRAS or NRAS mutation | C15–C26: digestive organs | To evaluate the safety, efficacy, and doses of a combination regimen | Onvansertib + SoC (FOLFIRI + Bevacizumab) vs. SoC | 2024–2027; USA |
| NCT06225999 | oxaliplatin | Single-group assignment open-label study | Phase 2 | Metastatic adenocarcinoma of the pancreas | C15–C26: digestive organs | To evaluate the efficacy and/or safety of a combination regimen | Irinotecan liposome injection in combination with oxaliplatin, 5-FU and LLV (levoisomer form of leucovorin) | 2024–2026; Japan |
| NCT06239532 | oxaliplatin | Single-center, single-arm, prospective study | Phase 2 | Unresectable intrahepatic cholangiocarcinoma | C15–C26: digestive organs | To evaluate the efficacy and/or safety of a combination regimen | Hepatic arterial infusion chemotherapy (HAIC = oxaliplatin + raltitrexed) sequential transcatheter arterial embolization (TAE) + tislelizumab + surufatinib | 2022–2025; China |
| NCT06256328 | oxaliplatin | Randomized, multicenter, double-blind study | Phase 2 | HER2- negative unresectable advanced or recurrent gastric cancer (including esophagogastric junction cancer) | C15–C26: digestive organs | To evaluate the efficacy and/or safety of a combination regimen | ONO-4578 + nivolumab + chemotherapy (including oxaliplatin) vs. placebo + nivolumab + chemotherapy (including oxaliplatin) | 2023–2027; Japan |
| NCT06429904 | oxaliplatin | Prospective, open-label, single-arm study | Phase 2 | Locally advanced pancreatic cancer | C15–C26: digestive organs | To evaluate the efficacy and/or safety of a combination regimen | Nimotuzumab + NALIRIFOX (liposomal irinotecan + oxaliplatin + leucovorin + fluorouracil) | 2024–2027; China |
| NCT06601504 | oxaliplatin | Randomized, controlled study parallel-group study | Phase 2 | Relapsed/refractory diffuse large B-cell lymphoma (R/R DLBCL) | C42: hematopoietic and reticuloendothelial systems | To evaluate safety, efficacy and pharmacokinetic characteristics of a combination | HMPL-760 + R-GemOx (rituximab, gemcitabine, oxaliplatin)) vs. placebo + R-GemOx | 2024–2026; China |
| NCT06667050 | oxaliplatin | Single-group assignment open-label study | Phase 2 | Locally advanced gastric cancer | C15–C26: digestive organs | To evaluate the efficacy and/or safety of a combination regimen | Chemotherapy + sintilimab + bevacizumab biosimilar | 2024–2027; China |
| NCT06681298 | oxaliplatin | Multicenter, two-arm, randomized study | Phase 2 | HER2-positive resectable esophagogastric junctional adenocarcinoma | C15–C26: digestive organs | To evaluate the efficacy and/or safety of a combination regimen | Nivolumab + FLOT + trastuzumab + surgical treatment vs. trastuzumab + surgical treatment + FLOT + trastuzumab | 2024–2029; China |
| NCT06766019 | oxaliplatin | Interventional single-group assignment open-label study | Phase 2 | Locally advanced pancreatic cancer | C15–C26: digestive organs | To evaluate the efficacy and/or safety of a combination regimen | Adebrelimab + irinotecan liposomal (II), oxaliplatin, and 5-FU/LV | 2024–2027; China |
| NCT06853769 | oxaliplatin | Interventional single-group assignment open-label study | Phase 1 | Locally advanced rectal cancer | C15–C26: digestive organs | To evaluate the efficacy and/or safety of a combination regimen | Anlotinib hydrochloride, penpulimab + chemotherapy (capecitabine plus oxaliplatin) | 2024–2027; China |
| NCT06939439 | oxaliplatin | Randomized, controlled study | Phase 3 | Stage III gastric or gastroesophageal junction adenocarcinoma ctDNA-MRD positive | C15–C26: digestive organs | To evaluate the efficacy and/or safety of a combination regimen | Tislelizumab + oxaliplatin + tegafur vs. oxaliplatin + tegafur | 2024–2029; China |
| NCT07020468 | oxaliplatin | Single-center, single-arm, prospective study | Phase 4 | Locally advanced colorectal cancer and upper rectal cancer | C15–C26: digestive organs | To evaluate the efficacy and/or safety of a combination regimen | Oxaliplatin + irinotecan liposome + 5-FU/LV | 2025–2027; China |
| NCT07024537 | oxaliplatin | Single-arm, single-center study | Phase 2 | Colorectal cancer liver metastases | C15–C26: digestive organs | To evaluate the efficacy and/or safety of a combination regimen | FOLFOXIRI-HAIP chemotherapy (including oxaliplatin) + fruquintinib | 2023–2026; China |
| NCT05248022 | nedaplatin | Prospective, multicenter, randomized, open-label, two-arm study | Not applicable | Patients with stage II/III NSCLC after failure of or intolerance to standard treatment | C30–C39: respiratory system and intrathoracic organs | To evaluate efficacy and safety | Drug-eluting bead bronchial arterial chemoembolization (DEB-BACE; cisplatin, carboplatin, or nedaplatin; drug-loaded microspheres containing vinorelbine, gemcitabine, irinotecan, or raltitrexed) plus sintilimab vs. DEB-BACE alone | 2024; China |
| NCT05351762 | nedaplatin | Prospective, open-label, single-arm study | Phase 2 | Resectable locally advanced head and neck tumors | Head and neck | To evaluate efficacy and safety | Nimotuzumab combined with neoadjuvant chemotherapy (nedaplatin) | 2022–2024; China |
| NCT05515315 | nedaplatin | Interventional study | Phase 2 | Locally unresectable esophageal squamous cell carcinoma (ESCC) | C15–C26: digestive organs | To evaluate the efficacy of tislelizumab combined with chemotherapy | Tislelizumab plus nedaplatin-based chemotherapy | 2022–2027; China |
| NCT05624099 | nedaplatin | Interventional study | Phase 2 | Advanced esophageal cancer | C15–C26: digestive organs | To evaluate efficacy and safety | Camrelizumab combined with chemoradiotherapy and camrelizumab combined with chemotherapy | 2023–2027; China |
| NCT05772377 | nedaplatin | Exploratory study | Phase 2 | FIGO stage IB3 and IIA2-IVA cervical cancer | C51–C58: female genital organs | To evaluate efficacy and safety of hydrochloride anlotinib combined with concurrent radiochemotherapy | Anlotinib plus TP chemotherapy followed by concurrent chemoradiotherapy with anlotinib (nedaplatin or cisplatin) | 2023–2025; China |
| NCT05872724 | nedaplatin | Prospective cohort study | Phase 2 | Early postoperative cervical cancer | C51–C58: female genital organs | To evaluate efficacy and safety | Chemoradiotherapy plus adjuvant chemotherapy and zimberelimab vs. small-pelvic-field chemoradiotherapy plus zimberelimab (nedaplatin or carboplatin) | 2023–2028; China |
| NCT05880082 | nedaplatin | Interventional study | Phase 2 | Esophageal cancer | C15–C26: digestive organs | To evaluate objective response and major pathological response rates | Tislelizumab, albumin-bound paclitaxel, carboplatin, or cisplatin or nedaplatin | 2023–2025; China |
| NCT05944809 | nedaplatin | Prospective study | Phase 2 | Esophageal cancer | C15–C26: digestive organs | To evaluate the protective effect of prophylactic thrombopoietin combined with bone marrow-sparing intensity-modulated radiotherapy | Bone marrow-sparing intensity-modulated radiotherapy containing nedaplatin or cisplatin | 2023–2024; China |
| NCT06010212 | nedaplatin | Single-center, single-arm study | Phase 1/2 | Advanced esophageal squamous cell carcinoma | C15–C26: digestive organs | To evaluate efficacy and safety | Fruquintinib, camrelizumab, liposomal paclitaxel, and nedaplatin | 2023–2026; China |
| NCT06055153 | nedaplatin | Single-arm, exploratory study | Phase 1/2 | Locally advanced/metastatic HER2 overexpression (IHC 2+/3+) esophageal squamous cell carcinoma | C15–C26: digestive organs | To explore the safety and efficacy | Vedicetuzumab combined with an immune checkpoint inhibitor (camrelizumab) and platinum-based first-line treatment (cisplatin/nedaplatin) | 2023–2025; China |
| NCT06102395 | nedaplatin | Prospective, open-label, multicenter study | Phase 3 | Locally advanced (LA) HNSCC | Head and neck | To evaluate the efficacy and safety of pembrolizumab | Pembrolizumab plus standard chemotherapy containing nedaplatin, cisplatin, or carboplatin | 2023–2030; China |
| NCT06127329 | nedaplatin | Prospective, single-arm, open-label study | Not applicable | Advanced Non-small-cell lung cancer (NSCLC) | C30–C39: respiratory system and intrathoracic organs | To evaluate efficacy and safety | Bronchial infusion chemotherapy combined with drug-loaded microsphere embolization of EqualSpheres | 2023–2026; China |
| NCT06170697 | nedaplatin/lobaplatin | Prospective, single-center, single-arm study | Phase 2 | Head and neck cancer | Head and neck | To evaluate the efficacy and safety of camrelizumab combined with concurrent chemoradiotherapy | Camrelizumab plus radiotherapy and cisplatin, carboplatin, lobaplatin, or nedaplatin | 2023–2025; China |
| NCT06186726 | nedaplatin | Prospective study | Phase 2 | Inoperable locally advanced or advanced primary thymic epithelial tumor | C30–C39: respiratory system and intrathoracic organs | To evaluate the efficacy of carbon-ion radiotherapy combined with chemotherapy | Carbon-ion radiotherapy (72 GyE in 18 fractions) combined with platinum-containing chemotherapy (etoposide or paclitaxel plus cisplatin, carboplatin, lobaplatin, or nedaplatin) | 2024–2029; China |
| NCT06190782 | nedaplatin/lobaplatin | Randomized, controlled, parallel-group study | Phase 3 | Oligometastatic squamous cell carcinoma | Not specified | To evaluate the efficacy and safety of adding local therapy | PD-1 inhibitor with or without chemotherapy plus local therapy vs. PD-1 inhibitor with or without chemotherapy alone (carboplatin, nedaplatin, oxaliplatin, or lobaplatin) | 2022–2027; China |
| NCT06364046 | nedaplatin | Prospective, single-center, randomized, open-label, two-arm study | Not applicable | SCLC | C30–C39: respiratory system and intrathoracic organs | To compare DEB-BACE plus serplulimab with conventional intravenous chemotherapy plus serplulimab | DEB-BACE plus serplulimab vs. conventional intravenous chemotherapy (cisplatin, carboplatin, or nedaplatin) plus serplulimab | 2024–2025; China |
| NCT06424899 | nedaplatin | Randomized, controlled, multicenter study | Phase 2 | Potentially operable stage III NSCLC | C30–C39: respiratory system and intrathoracic organs | To evaluate efficacy and safety | Adebrelimab in combination with chemotherapy after 3 cycles as neoadjuvant therapy and surgery or chemoradiotherapy based on MDT compared with adebrelimab after chemoradiotherapy (cisplatin, carboplatin or nedaplatin) | 2024–2027; China |
| NCT06508229 | nedaplatin | Multicenter, single-arm study | Phase 2 | Locally advanced resectable esophageal squamous cell carcinoma | C15–C26: digestive organs | To evaluate efficacy and safety | Adebrelimab combined with albumin-bound paclitaxel and nedaplatin as neoadjuvant therapy | 2024–2028; China |
| NCT06569459 | nedaplatin | Randomized, controlled, single-center, two-arm study | Phase 2 | Locally advanced esophageal squamous cell carcinoma that is not resectable | C15–C26: digestive organs | To evaluate efficacy and safety | Chemoradiotherapy containing carboplatin or nedaplatin plus immunotherapy, with or without trilaciclib | 2024–2026; China |
| NCT06775275 | nedaplatin | Randomized study | Phase 2 | Resectable Stage II-IIIB (T3N2) NSQ-NSCLC | C30–C39: respiratory system and intrathoracic organs | To evaluate efficacy and safety of adebrelimab in combination with chemotherapy with or without bevacizumab | Adebrelimab; bevacizumab; platinum-based chemotherapy (platinums include carboplatin, cisplatin and nedaplatin) | 2024–2029; China |
| NCT06953323 | nedaplatin | Multicenter, three-part, open-label, non-randomized study | Phase 1/2 | Advanced solid tumors | Not specified | To preliminarily evaluate the safety, tolerability, pharmacokinetics, and efficacy of WJB001 combination therapy | WJB001 in combination with paclitaxel or albumin-bound paclitaxel; carboplatin or nedaplatin; paclitaxel plus carboplatin or nedaplatin; niraparib; bevacizumab; JS207 or toripalimab; or paclitaxel plus carboplatin or nedaplatin and JS207 or toripalimab | 2025–2029; China |
| NCT07015489 | nedaplatin | Prospective, single-center study | Phase 2 | Esophageal cancer | C15–C26: digestive organs | To evaluate efficacy and safety | Camrelizumab plus induction chemotherapy containing carboplatin or nedaplatin | 2022–2025; China |
| NCT07019181 | nedaplatin | Single-arm study | Phase 2 | Squamous esophageal cancer | C15–C26: digestive organs | To evaluate the pathological complete response rate | Tislelizumab + nedaplatin | 2025–2026; China |
| NCT07046221 | nedaplatin | Prospective, open-label, multicenter study | Phase 2 | Resectable esophageal squamous cell carcinoma | C15–C26: digestive organs | To evaluate non-inferiority | Tislelizumab plus chemotherapy containing cisplatin, carboplatin, or nedaplatin | 2025–2030; China |
| NCT07284836 | nedaplatin | Interventional study | Phase 2 | Breast cancer patients with liver metastases after failure of standard treatment | C50: breast | To evaluate the efficacy | Albumin-bound paclitaxel + nedaplatin | 2025–2028; China |
| NCT07374003 | nedaplatin | Single-arm study | Phase 2 | Locally advanced resectable esophageal squamous cell carcinoma | C15–C26: digestive organs | To evaluate efficacy and safety | Ipromlimab combined with tuvonralimab, plus albumin-bound paclitaxel and nedaplatin, as neoadjuvant therapy | 2025–2029; China |
| NCT04845490 | lobaplatin | Prospective, randomized, comparative study | Phase 2 | Advanced colorectal cancer | C15–C26: digestive organs | To compare the therapeutic effects of mitomycin and lobaplatin | Hyperthermic intraperitoneal chemotherapy (HIPEC) (with mitomycin) vs. hyperthermic intraperitoneal chemotherapy (HIPEC) (with lobaplatin) vs. hyperthermic intraperitoneal therapy (HIPET) (no drug) | 2022–2026; China |
| NCT06714383 | lobaplatin | Not reported | Phase 2 | Neoadjuvant treatment of stage II/III classic osteosarcoma | C40–C41: bones, joints, and articular cartilage | To evaluate the clinical application value in bone marrow protection | Trilaciclib in combination with pirarubicin and lobaplatin. Neoadjuvant therapy of adding trilaciclib to pirarubicin and lobaplatin vs. neoadjuvant therapy of pirarubicin and lobaplatin | 2024–2025; China |
| NCT05766605 | lobaplatin | Interventional study | Phase 3 | Liver cancer | C15–C26: digestive organs | To clarify the clinical feasibility of patient-derived organoid xenograft results for guiding interventional chemotherapy after surgery for primary liver cancer | Test group (postoperative conventional treatment (lobaplatin/cisplatin/oxaliplatin) + precise transarterial chemoembolization) vs. control group (postoperative conventional treatment + empirical transarterial chemoembolization with doxorubicin) | 2023–2026; China |
| NCT05701436 | lobaplatin | Interventional study | Phase 3 | Liver cancer | C15–C26: digestive organs | To evaluate the clinical feasibility of three-dimensional histoculture drug-response assay results for guiding interventional chemotherapy | Test group received postoperative conventional treatment (lobaplatin/cisplatin/oxaliplatin) combined with precise transarterial chemoembolization based on 3D-HDRA results. vs. The control group received postoperative conventional treatment combined with empirical transarterial chemoembolization | 2022–2026; China |
| NCT06169410 | lobaplatin | Interventional study | Phase 4 | Advanced gastric cancer | C15–C26: digestive organs | To evaluate efficacy and safety | Ramucirumab combined with nab-paclitaxel, lobaplatin and S-1 in neoadjuvant and conversion therapy: Experimental: ramucirumab combined with lobaplatin, S-1 and nab-paclitaxel vs. active comparator: lobaplatin combined with S-1 and nab-paclitaxel | 2023–2025; China |
| NCT06198465 | lobaplatin | Prospective, open-label, single-center study | Phase 2 | Esophageal and esophagogastric junction cancer | C15–C26: digestive organs | To evaluate efficacy and safety | Adebrelimab plus paclitaxel, lobaplatin, and fluorouracil | 2024–2025; China |
| NCT05780294 | lobaplatin | Randomized, double-blind, multicenter study | Phase 3 | Nasopharyngeal carcinoma | C00–C14: lip, oral cavity, and pharynx | To investigate the efficacy and safety of leucogen | Control: 5-FU, lobaplatin, and placebo; experimental: 5-FU, lobaplatin, and leucogen | 2023–2029; China |
| NCT06688175 | lobaplatin | Prospective, multicenter, single-arm, open-label study | Phase 2 | Elderly patients with nasopharyngeal carcinoma | C00–C14: lip, oral cavity, and pharynx | To investigate the efficacy and safety | Intensity-modulated radiotherapy + lobaplatin-based concurrent chemotherapy | 2024–2027; China |
| NCT07093931 | lobaplatin | Randomized, controlled study | Phase 3 | Stage II / III HER2 positive breast cancer | C50: breast | To evaluate efficacy, safety, and tolerability | Lobaplatin vs. carboplatin as neoadjuvant therapy | 2025–2030; China |
| NCT06609850 | lobaplatin | Interventional study | Not applicable | Hepatocellular carcinoma (HCC) at BCLC B stage | C15–C26: digestive organs | To evaluate ablation combined with TACE and lenvatinib for intermediate-stage hepatocellular carcinoma | TACE (including lobaplatin) plus lenvatinib vs. TACE plus lenvatinib and ablation | 2024–2028; China |
| NCT06740370 | lobaplatin | Prospective multicenter study | Not applicable | Hepatocellular carcinoma (HCC) with spontaneous rupture | C15–C26: digestive organs | To evaluate TACE combined with lenvatinib and a PD-1 inhibitor as first-line therapy | TACE (including lobaplatin) plus lenvatinib and PD-1 inhibitor | 2024–2026; China |
| NCT06898398 | lobaplatin | Observational study | Not applicable | Huge hepatocellular carcinoma (HCC) | C15–C26: digestive organs | To evaluate neoadjuvant TACE before liver resection in patients with large hepatocellular carcinoma | TACE containing lobaplatin followed by surgery vs. surgery alone | 2025; China |
| NCT05472896 | dicycloplatin | Open-label, randomized, parallel-group, multicenter study | Phase 3 | Hepatocellular carcinoma | C15–C26: digestive organs | To evaluate the efficacy and safety of TP21 (dicycloplatin) injection for TACE | Conventional TACE with epirubicin vs. conventional TACE with TP21 (dicycloplatin) | 2022–2024; China |
| NCT05104736 | PT-112 | Open-label trial | Phase 2 | Relapsed or refractory thymic epithelial tumors | Not specified | To determine the objective response rate | PT-112 monotherapy | 2022–2028; USA |

***Abbreviations:*** *5-FU, 5-fluorouracil; AC, doxorubicin plus cyclophosphamide; BCG, Bacillus Calmette–Guérin; BCLC, Barcelona Clinic Liver Cancer; BT, brachytherapy; CAPOX, capecitabine plus oxaliplatin; CAR-T, chimeric antigen receptor T-cell; CCRT, concurrent chemoradiotherapy; CNS, central nervous system; CPI, checkpoint inhibitor; CRLM, colorectal liver metastases; CRT, chemoradiotherapy; DCF, docetaxel, cisplatin, and 5-fluorouracil; DEB-BACE, drug-eluting bead bronchial arterial chemoembolization; DLBCL, diffuse large B-cell lymphoma; DNA, deoxyribonucleic acid; EBRT, external-beam radiotherapy; EC, esophageal cancer; EGFR, epidermal growth factor receptor; ES-SCLC, extensive-stage small-cell lung cancer; ESCC, esophageal squamous-cell carcinoma; FIGO, International Federation of Gynecology and Obstetrics; FLOT, 5-fluorouracil, leucovorin, oxaliplatin, and docetaxel; FOLFIRI, 5-fluorouracil, leucovorin, and irinotecan; FOLFOX, 5-fluorouracil, leucovorin, and oxaliplatin; FOLFOXIRI, 5-fluorouracil, leucovorin, oxaliplatin, and irinotecan; GC, gastric cancer; GEJ, gastroesophageal junction; GP, gemcitabine plus cisplatin; HAIC, hepatic arterial infusion chemotherapy; HAIP, hepatic arterial infusion pump; HCC, hepatocellular carcinoma; HDRA, histoculture drug-response assay; HER2, human epidermal growth factor receptor 2; HIPEC, hyperthermic intraperitoneal chemotherapy; HIPET, hyperthermic intraperitoneal therapy; HNSCC, head and neck squamous-cell carcinoma; IC, induction chemotherapy; ICD-O, International Classification of Diseases for Oncology; ICI, immune checkpoint inhibitor; IHC, immunohistochemistry; LANPC, locoregionally advanced nasopharyngeal carcinoma; LLV, levoleucovorin; LV, leucovorin; MDT, multidisciplinary team; MIRV, mirvetuximab soravtansine; MRD, minimal residual disease; MSS, microsatellite stable; NALIRIFOX, liposomal irinotecan, 5-fluorouracil, leucovorin, and oxaliplatin; NPC, nasopharyngeal carcinoma; NSCLC, non-small-cell lung cancer; NSQ-NSCLC, non-squamous non-small-cell lung cancer; PD-1, programmed cell death protein 1; PD-L1, programmed death-ligand 1; PDAC, pancreatic ductal adenocarcinoma; R-ICE, rituximab, ifosfamide, carboplatin, and etoposide; RC, radical cystectomy; SCLC, small-cell lung cancer; SOC, standard of care; SOX, S-1 plus oxaliplatin; TACE, transarterial chemoembolization; TAE, transarterial embolization; TIL, tumor-infiltrating lymphocyte; TKI, tyrosine kinase inhibitor; TP, paclitaxel plus cisplatin; TPC, taxane, platinum, and capecitabine; TPS, tumor proportion score; TRT, thoracic radiotherapy; UK, United Kingdom; USA, United States of America; wTAX, weekly paclitaxel; XELOX, capecitabine plus oxaliplatin.*

## **Drug utilization data**

Number of packages

|  | Cisplatin | Oxaliplatin | Carboplatin |
| --- | --- | --- | --- |
| 1.2021 | 1364.71 | 1913.4 | 3115.99 |
| 2.2021 | 1372.12 | 1930.54 | 3003.03 |
| 3.2021 | 1414.45 | 1950.21 | 3195.35 |
| 4.2021 | 1521.29 | 1939.36 | 3272.26 |
| 5.2021 | 1295.51 | 1709 | 2754.35 |
| 6.2021 | 1525.9 | 2098.55 | 3256.41 |
| 7.2021 | 1702.16 | 2253.86 | 3342.4 |
| 8.2021 | 1791.35 | 2188.23 | 3325.38 |
| 9.2021 | 1639.03 | 2175.11 | 3464.8 |
| 10.2021 | 1726.87 | 2236.2 | 3514.26 |
| 11.2021 | 1532.72 | 2017.15 | 3210.28 |
| 12.2021 | 1473.41 | 2031.08 | 3412.66 |
| 1.2022 | 1399.52 | 1959.58 | 2948.36 |
| 2.2022 | 1416.53 | 1941.04 | 2886.66 |
| 3.2022 | 1601.21 | 2218.49 | 3268.18 |
| 4.2022 | 1546.66 | 2099.04 | 3409.91 |
| 5.2022 | 1528.59 | 2196.11 | 3067.96 |
| 6.2022 | 1547.83 | 2328.95 | 3482.52 |
| 7.2022 | 1634.26 | 2364.97 | 3602.69 |
| 8.2022 | 1581.16 | 2384.9 | 3622.46 |
| 9.2022 | 1449.45 | 2272.74 | 3503.45 |
| 10.2022 | 1462.66 | 2327.69 | 3529.72 |
| 11.2022 | 1635.23 | 2326.28 | 3565.29 |
| 12.2022 | 1451.4 | 2205.14 | 3296.97 |
| 1.2023 | 1542.21 | 2322.42 | 3416.35 |
| 2.2023 | 1393.38 | 2354.54 | 3346.72 |
| 3.2023 | 1463 | 2763.15 | 3940.67 |
| 4.2023 | 1236.42 | 2393.2 | 3639.49 |
| 5.2023 | 1233.55 | 2419.94 | 3561.84 |
| 6.2023 | 1270.63 | 2849.99 | 4064.07 |
| 7.2023 | 1617.79 | 2792.65 | 3946.32 |
| 8.2023 | 1357.66 | 2892.98 | 4061.63 |
| 9.2023 | 1508.93 | 2775.7 | 3860.94 |
| 10.2023 | 1205.6 | 2750.24 | 3935.01 |
| 11.2023 | 1322.99 | 2927.76 | 4053.87 |
| 12.2023 | 1191.43 | 2495.65 | 3634.84 |
| 1.2024 | 1542.21 | 2322.42 | 3416.35 |
| 2.2024 | 1393.38 | 2354.54 | 3346.72 |
| 3.2024 | 1463 | 2763.15 | 3940.67 |
| 4.2024 | 1236.42 | 2393.2 | 3639.49 |
| 5.2024 | 1233.55 | 2419.94 | 3561.84 |
| 6.2024 | 1270.63 | 2849.99 | 4064.07 |
| 7.2024 | 1617.79 | 2792.65 | 3946.32 |
| 8.2024 | 1357.66 | 2892.98 | 4061.63 |
| 9.2024 | 1508.93 | 2775.7 | 3860.94 |
| 10.2024 | 1205.6 | 2750.24 | 3935.01 |
| 11.2024 | 1322.99 | 2927.76 | 4053.87 |
| 12.2024 | 1191.43 | 2495.65 | 3634.84 |

**Figure S1**. Number of packages of Pt-based drugs per month (2021-2024) used in Bulgaria

## **Neoplasms treated with Pt-drugs**

|  | C00-C14 LIP, ORAL CAVITY AND PHARYNX | C15-C26 DIGESTIVE ORGANS | C30-C39 RESPIRATORY SYSTEM AND INTRATORACIC ORGANS | C40-C41 BONES, JOINTS and ARTICULAR CARTILAGE | C42 HEMATOPOIETIC AND RETICULOENDOTHELIAL SYSTEMS | C44 SKIN | C47 PERIPHERAL NERVES AND AUTONOMIC NERVOUS SYSTEM | C48 RETROPERITONEUM AND PERITONEUM | C49 CONNECTIVE, SUBCUTANEOUS AND OTHER SOFT TISSUES | C50 BREASТ | C51-C58 FEMALE GENITAL ORGANS | C60-C63 MALE GENITAL ORGANS | C64-C68 URINARY TRACT | C69-C72 EYE, BRAIN AND OTHER PARTS OF CENTRAL NERVOUS SYSTEM | C73-C75 THYROID AND OTHER ENDOCRINE GLANDS | С76 OTHER AND ILL-DEFINED SITES | C77 LYMPH NODES | C80 UNKNOWN PRIMARY SITE |
| --- | --- | --- | --- | --- | --- | --- | --- | --- | --- | --- | --- | --- | --- | --- | --- | --- | --- | --- |
| 1.01.21 cisplatin | 69 | 93 | 113 | 1 | 16 |  |  |  |  | 9 | 91 | 31 | 33 | 1 |  | 4 | 12 | 4 |
| 1.01.21 carboplatin | 17 | 23 | 284 |  | 4 | 4 |  | 3 | 1 | 58 | 396 | 9 | 28 | 3 |  | 7 | 10 | 6 |
| 1.01.21 oxaliplatin |  | 952 |  |  | 1 |  |  |  |  |  | 1 |  | 1 |  |  |  | 10 | 3 |
| 1.12.21 cisplatin | 77 | 101 | 143 |  |  | 12 |  |  | 2 | 20 | 85 | 40 | 35 |  |  | 1 | 3 | 2 |
| 1.12.21 carboplatin | 17 | 20 | 321 | 1 | 3 | 5 |  | 7 | 1 | 75 | 407 | 9 | 23 |  |  | 6 | 6 | 6 |
| 1.12.21 oxaliplatin |  | 1109 | 2 |  | 1 |  |  |  |  | 1 |  |  | 1 |  |  | 1 | 7 |  |
| 1.01.22 cisplatin | 77 | 76 | 37 | 1 | 16 | 1 |  |  | 2 | 22 | 22 | 45 | 30 |  |  | 3 | 5 | 4 |
| 1.01.22 carboplatin | 18 | 16 | 54 | 1 | 6 | 3 |  | 4 |  | 65 | 55 | 10 | 19 |  | 1 | 2 | 7 | 4 |
| 1.01.22 oxaliplatin |  | 1125 | 2 |  |  |  |  |  |  | 1 |  |  |  |  |  |  | 9 | 2 |
| 1.12.22 cisplatin | 95 | 92 | 36 |  | 10 | 4 |  | 1 | 1 | 12 | 25 | 28 | 42 |  | 1 | 5 | 3 | 2 |
| 1.12.22 carboplatin | 13 | 17 | 60 |  | 7 | 4 |  | 2 |  | 98 | 57 | 11 | 43 |  |  | 4 |  | 3 |
| 1.12.22 oxaliplatin |  | 1153 |  |  | 1 |  |  | 1 |  | 2 |  |  |  |  |  | 1 |  | 2 |
| 1.01.23 cisplatin | 83 | 132 | 123 | 1 | 19 | 2 |  | 2 | 1 | 17 | 99 | 37 | 52 | 2 | 2 | 6 | 1 | 4 |
| 1.01.23 carboplatin | 13 | 21 | 365 |  | 6 | 6 |  | 4 | 1 | 102 | 446 | 9 | 38 |  |  | 9 | 1 | 10 |
| 1.01.23 oxaliplatin |  | 1233 |  |  | 1 |  |  | 1 |  | 2 |  |  |  |  |  |  |  | 1 |
| 1.12.23 cisplatin | 67 | 101 | 105 |  | 13 |  |  |  | 1 | 7 | 75 | 22 | 38 | 1 | 4 | 3 | 5 |  |
| 1.12.23 carboplatin | 34 | 46 | 381 | 1 | 3 | 8 |  | 4 |  | 97 | 489 | 7 | 51 | 1 | 1 | 4 |  | 9 |
| 1.12.23 oxaliplatin |  | 1365 | 1 |  | 1 |  |  |  |  |  | 2 |  |  |  |  |  |  | 3 |
| 1.01.24 cisplatin | 59 | 93 | 88 |  | 13 | 3 |  | 1 | 2 | 3 | 61 | 21 | 26 | 2 |  | 5 |  | 6 |
| 1.01.24 carboplatin | 50 | 61 | 443 |  | 1 | 7 | 4 |  |  | 105 | 508 | 13 | 72 | 1 | 1 | 4 |  | 9 |
| 1.01.24 oxaliplatin |  | 1557 |  |  | 5 |  |  |  |  |  | 2 | 1 |  |  |  |  |  | 2 |
| 1.12.24 cisplatin | 78 | 120 | 121 |  | 5 | 2 |  | 1 | 1 | 6 | 82 | 37 | 39 | 1 | 1 | 4 |  | 10 |
| 1.12.24 carboplatin | 30 | 43 | 402 |  | 12 | 4 |  | 4 |  | 151 | 404 | 10 | 41 |  | 1 | 7 |  | 10 |
| 1.12.24 oxaliplatin |  | 1334 | 1 |  |  |  |  | 1 |  |  |  |  | 1 |  |  |  |  | 3 |

**Figure S2**. Neoplasms (classified according to ICD-O) treated with Pt-drugs in Bulgaria (January and December for the study period)

## **Pt-NHC and multifunctional Pt(IV) complexes papers**

Number of articles

|  | Pt-NHC compounds | multifunctional Pt(IV) prodrugs |
| --- | --- | --- |
| 2010 | 2 |  |
| 2011 | 3 | 1 |
| 2013 | 4 | 1 |
| 2014 | 2 | 1 |
| 2015 | 3 | 5 |
| 2016 | 7 | 4 |
| 2017 | 2 | 6 |
| 2018 | 5 | 10 |
| 2019 | 3 | 11 |
| 2020 | 4 | 11 |
| 2021 | 3 | 10 |
| 2022 | 1 | 9 |
| 2023 | 5 | 13 |
| 2024 | 3 | 10 |
| 2025 | 9 | 14 |

**Figure S3.** Number of publications indexed in PubMed referring to Pt-NHC and multifunctional Pt(IV) anticancer compounds (2010-2025). The searches were performed with PubMed (see Table S1 for the search terms)
